# Fourier convolutional decoder: reconstructing solar flare images via deep learning

**DOI:** 10.1007/s00521-025-11283-6

**Published:** 2025-05-27

**Authors:** Merve Selcuk-Simsek, Paolo Massa, Hualin Xiao, Säm Krucker, André Csillaghy

**Affiliations:** 1 Institute for Data Science, School of Computer Science, University of Applied Sciences and Arts Northwestern Switzerland FHNW, Bahnhofstrasse 6, 5210 Windisch, Switzerland; 2 Space Sciences Laboratory, University of California, 7 Gauss Way, Berkeley, CA 94720 USA

**Keywords:** Image reconstruction, Deep learning, Autoencoder, Solar flare imaging, X-ray imaging

## Abstract

Reconstructing images from observational data is a complex and time-consuming process, particularly in astronomy, where traditional algorithms like CLEAN require extensive computational resources and expert interpretation to distinguish genuine features from artifacts, especially without ground truth data. To address these challenges, we developed the Fourier convolutional decoder (FCD), a custom-made overcomplete autoencoder trained on simulated data with available ground truth. This enables the network to generate outputs that closely approximate expected ground truth. The model’s versatility was demonstrated on both simulated and observational datasets, with a specific application to data from the spectrometer/telescope for imaging X-rays (STIX) on the solar orbiter. In the simulated environment, FCD’s performance was evaluated using multiple-image reconstruction metrics, demonstrating its ability to produce accurate images with minimal artifacts. For observational data, FCD was compared with benchmark algorithms, focusing on reconstruction metrics related to Fourier components. Our evaluation found that FCD is the fastest imaging method, with runtimes on the order of milliseconds. Its computational cost is comparable to the most efficient reconstruction algorithm and 280${\times }$ faster than the slowest imaging method for single-image reconstruction on a CPU. Additionally, its runtime can be reduced by an order of magnitude for multiple-image reconstruction on a GPU. FCD outperforms or matches state-of-the-art methods on simulated data, achieving a mean MS-SSIM of 0.97, LPIPS of 0.04, PSNR of 35.70 dB, a Dice coefficient of 0.83, and a Hausdorff distance of 5.08. Finally, on experimental STIX observations, FCD remains competitive with top methods despite reduced performance compared to simulated data.

## Introduction

Indirect imaging is a technique employed when direct visual observation, such as through a camera, is not possible. Instead, data are collected through alternative methods, such as interferometry, tomography, or magnetic resonance imaging, where it is represented as frequency components in the Fourier domain. Reconstruction of images from these Fourier components is a common challenge in many scientific and engineering domains, including medical imaging [1], signal processing [2], and astrophysics [3]. Traditional algorithms often involve an inverse Fourier transform followed by additional algorithms to handle noise, artifacts, and, especially, incomplete data. These processes, typical of inverse problem formulations, can be computationally intensive and may introduce reconstruction errors, particularly in complex scenarios.

In response to these challenges, we propose a machine learning-based approach, specifically utilizing deep learning. We introduce a novel autoencoder [4] architecture designed to process Fourier components directly, eliminating the need for an explicit inverse Fourier transform and subsequent algorithms. This approach leverages the strengths of deep learning to produce high-quality reconstructed images while reducing computational overhead. Further, this deep learning model is implemented in a way that requires very limited memory resources to be stored and run: 3.74 MiB for the model weights and 11.11 MiB for the entire model^1^. Given its compact size, the model is highly suitable for deployment on edge devices and systems with limited computational resources. In principle, this model could even be stored and executed on-board space instruments, making it an ideal candidate for real-time data processing in resource-constrained environments.

In the realm of astronomy, machine learning techniques have been increasingly utilized for image reconstruction from various input types, demonstrating their versatility in enhancing astronomical observations and analyses. Deep learning, in particular, has seen growing applications in solar physics, addressing tasks such as solar image denoising [5, 6] and image generation [7]. While deep learning has also been explored for image reconstruction in radio astronomy [8, 9] and more recently in solar X-ray astronomy [10], our approach differs by employing a highly compact model that directly processes Fourier components.

In this study, we use a custom-built autoencoder, referred to as the Fourier convolutional decoder (FCD), to reconstruct solar flare images from the spectrometer/telescope for imaging X-rays (STIX) data. The custom autoencoder we present offers a novel approach in solar physics, specifically for imaging solar flares, by processing Fourier components to reconstruct images. Our approach aims to efficiently reconstruct images with minimal artifacts^2^ that closely approximate the ground truth in the simulated data. We subsequently test our approach using both simulated and observational data from STIX, applying two different sets of metrics.

The STIX image reconstruction process is particularly challenging due to the highly restricted number of Fourier components measured by the instrument, which are insufficient to fully constrain the imaging solution. From a mathematical perspective, this limitation means that the problem has infinite possible solutions. Therefore, every image reconstruction method relies on a priori knowledge to select a specific solution from these infinite possibilities that fit the available data with comparable accuracy. Traditionally, this prior information is mathematically formalized through a sparsity property with respect to appropriate dictionaries [11, 12] or a maximum entropy principle [13]. Unlike state-of-the-art methods for STIX image reconstruction, our proposed FCD *learns* an implicit prior directly from the training data, eliminating the need to explicitly enforce “smoothness” or suppress artifacts. This approach provides a key advantage over existing methods, as it removes the requirement to mathematically define a regularized solution and to integrate this definition into the reconstruction process. Additionally, FCD is a parameter-free imaging method, making it easily accessible to non-experts in solar physics. All these advantages are made possible by the creation of a dataset of image data pairs for training the model.

We summarize the main contributions of this work as follows:

- *Introduction of a deep learning approach for image reconstruction from solar X-ray Fourier components:* For the first time, we propose a deep learning-based method for reconstructing images from Fourier components measured by the STIX instrument. While the concept behind our approach aligns with the method recently presented in [10] for hard X-ray imager (HXI) data, our model is significantly smaller, enabling potential on-board space application. Additionally, unlike the model in [10], our FCD directly processes Fourier components as input, rather than raw detector data, i.e., unprocessed data before frequency transformation.
- *Training and testing on simulated and observational STIX data:* We train the proposed FCD on a simulated dataset and evaluate its performance on both simulated data and actual STIX observations. Our results demonstrate that the FCD outperforms existing methods on simulated data and ranks among the top-performing algorithms for observational STIX data.
- *Computational efficiency assessment:* We assess the computational efficiency of the FCD, showing it to be the most efficient imaging method for analyzing STIX data.

The remainder of the paper is organized as follows. Section 2 overviews the domain-specific aspects relevant to this research, including detailed information about STIX, the process and challenges of solar flare image reconstruction, and the existing algorithms in this field. Furthermore, Subsection 2.3 in this section details the datasets—both observational and simulated—used in our experiments. Section 3 outlines our approach and introduces the autoencoder architecture. Section 4 presents the algorithmic framework and describes the computational steps of our reconstruction method. Section 5 defines the metrics sets we use in this work to evaluate our approach, while Sect. 6 covers the evaluation by presenting the experiments, results, and discussion. Finally, we draw our conclusions in Sect. 7.

## Solar physics data: instrumentation, acquisition, preparation, and processing

A solar flare occurs when magnetic field lines near sunspots, areas of intense magnetic activity, become twisted and then suddenly reconnect in a lower-energy configuration, releasing energy previously stored in the magnetic field. This process accelerates particles to relativistic energy levels and heats plasma to temperatures of up to 50 million Kelvin [14]. Observing solar flares has been an integral part of solar astronomy, as they provide valuable insights into solar activity and help in preparing for their potential impact on Earth, such as disruptions to communication systems and power grids.

Solar orbiter is a spacecraft launched in 2020 and operated by the European Space Agency. It is designed to study the Sun and its heliosphere. It carries a suite of ten specialized instruments to gather comprehensive data on solar activity [15]. Among these, the spectrometer/telescope for imaging X-rays (STIX) instrument operates in the X-ray range, specifically observing hard X-rays, i.e., higher energy and shorter wavelength emissions, from solar flares [16]. STIX uses an indirect imaging technique, reconstructing images from measurements rather than capturing them directly. Since the beginning of the nominal phase in January 2021, it has been continuously collecting data, and the STIX data center now holds a repository of more than 50,000 solar flare events [17]. This extensive dataset includes various types of measurements, such as light curves, spectra, and images, depending on the specific needs of the study. In this work, we focus on the image representations of these solar flare events, often referred to as “maps” in solar physics.

In the context of solar physics, the term *map* is commonly used to refer to a two-dimensional (2D) image array representing the distribution of a physical quantity on the Sun. In the case of STIX, the map represents the spatial distribution of the intensity of the X-ray radiation emitted by a solar flare. In contrast, in machine learning and image processing, the same data are referred to as an *image*, focusing on the pixel-based representation of visual information. For consistency, we retain the respective terminology in the relevant sections of the paper.

**Fig. 1:**
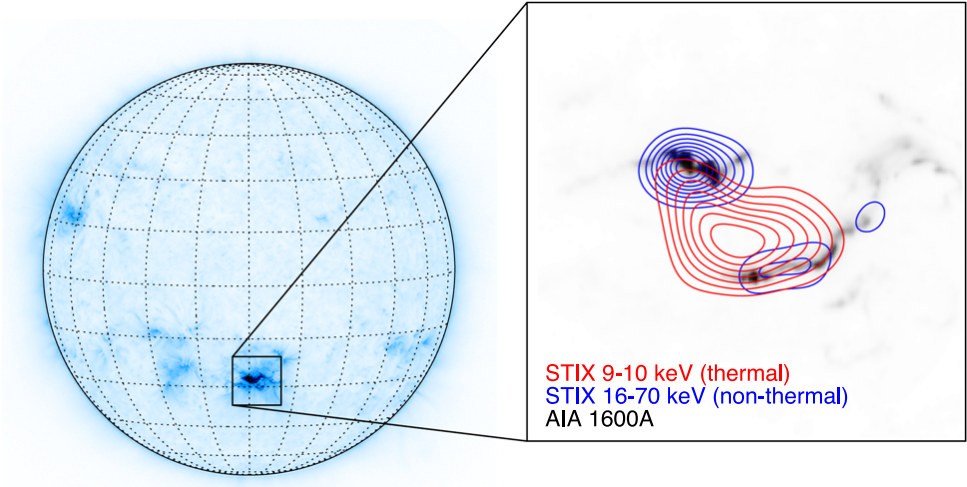
October 28 2021 flaring event observed by SDO/AIA and by STIX. The left panel shows a full-disk image registered by SDO/AIA in the 131 Å EUV channel. The insert to the right showcases the solar flare images reconstructed from the X-ray data collected by STIX at the thermal (9–10 keV) and non-thermal (16–70 keV) energy ranges overlaid to an UV 1600Å image from SDO/AIA

Figure 1 shows a solar flare that occurred on October 28 2021 at around 15 UT. The event was observed in extreme ultraviolet (EUV) wavelengths by the atmospheric imaging assembly (AIA) on board of the solar dynamics observatory (SDO) [SDO/AIA 18] and at different energies by STIX. The left panel shows a full-disk map recorded by SDO/AIA in the 131 Å (angstrom) channel, where an angstrom is a unit of length used to measure the wavelength of light, specifically in the ultraviolet range. This map is plotted with a synthetic color map representing the intensity of the EUV emission: dark areas correspond to high intensity. The flare location, appearing as a dark spot, is surrounded by a box. The right panel shows a zoom in of the flaring site. Specifically, three maps are overlaid in this panel. The grayscale image in the background is a map recorded by SDO/AIA in the 1600 Å channel, which shows two areas of intense emission as black spots. The red and blue contours represent the contour levels of the STIX maps that are reconstructed from data recorded in the 9–10 kilo-electronvolt (keV) range and in the 16–70 keV range, respectively, where keV is a unit of energy commonly used to describe X-rays. The red contours represent the intensity of the thermal emission, i.e., X-rays produced by plasma at a temperature of several million degrees [19]. The blue contours correspond to the intensity of the non-thermal emission. The latter is produced when the electrons accelerated by the reconfiguration of the magnetic field collide with the dense layers of the solar surface [20]. Note that given the limitations of the STIX imaging system, the reconstructed STIX images do not present fine structures or details. Rather, they usually consist of one or more “blobs” located where the X-ray emission is most intense. In this work, we focus on reconstructed STIX images as visualized in the right panel of Fig. 1.

Throughout the paper, we sample and display reconstructed STIX images, with links to their corresponding solar flare data provided in Appendix B. Bear in mind that the STIX instrument observes the Sun as ${90}^{\circ }$-rotated since it is mounted on the side of solar orbiter. Therefore, to properly display the reconstructed STIX images on the solar disk, a rotation of ${90}^{\circ }$ needs to be applied, as well as a correction to account for the instrument pointing and the spacecraft roll angle. However, in the remainder of the paper, we will display the reconstructed images as conceived in the native reference frame of the instrument and no correction will be applied.

### Instrumentation: the STIX instrument and the challenges of image reconstruction

Since hard X-rays are highly energetic, focusing them on a sensor, as is done with other wavelengths, is technically challenging. Hard X-ray focusing optics (e.g., [21]) has not been an option for a solar orbiter X-ray imager due to the limited resources available on Solar Orbiter. To address this issue, the STIX instrument applies an indirect imaging technique based on the use of *sub-collimators*, i.e., pairs of tungsten grids located in front of X-ray detectors [16]. The radiation emitted by solar flares and transmitted through the grids casts a Moiré pattern [22, 23] on the detector surface. This pattern is sensitive to the location and morphology of the X-ray source(s) and encodes information on a specific *visibility*, i.e., a 2D Fourier component of the solar flare X-ray signal. Therefore, the measurements provided by the STIX detectors consist of a limited number ($\sim$30) of visibilities corresponding to a fixed set of frequencies on the Fourier (*u*, *v*) plane, as detailed in [24]. To reconstruct the image of the flaring X-ray sources from STIX measurements, it is then necessary to solve an inverse problem from limited Fourier data. Further details about solar flare imaging in the hard X-ray domain can be found in [25].

Imaging for STIX presents two main challenges. First, the solution of the inverse problem is not unique due to the limited number of visibilities measured by the instrument. Second, the experimental visibility values are derived from counting of X-ray photons performed by the detectors, and the counting process is affected by Poisson noise and inaccuracies in the applied calibration steps. Therefore, the visibility values are affected by statistical noise and systematic effects, which can give origin to spurious artifacts in the retrieved images that are amplified during the reconstruction process. To address these two issues, several image reconstruction techniques have been implemented in recent decades in the context of solar flare X-ray imaging, many of which are inspired by developments in the similar field of radio interferometry [26].

Having access to multiple imaging algorithms enables the comparison of STIX images obtained through different techniques. In this way, it is possible to understand if a specific feature in a reconstructed image is an artifact introduced by the adopted imaging method, or if it is consistent between the different imaging techniques and, hence, real. Given the non-uniqueness of the solution for STIX imaging, no image reconstruction method is consistently optimal. Every imaging technique comes with its own strengths and limitations; thus, it is crucial to keep developing new algorithms, which could outperform existing methods under specific circumstances. Further, the implemented imaging techniques must provide regularized solutions with limited amount of artifacts by avoiding overfitting of noisy data and by introducing a priori information on the sought solution. Currently, five algorithms are available for reconstructing solar flare images using the analysis software implemented in the interactive data language (IDL) [24, 27]. The goal of this work is to develop a deep learning approach for STIX imaging.

### Current image reconstruction algorithms

In this research, we focus on three algorithms—VIS_FWDFIT, MEM_GE, and CLEAN—that have been implemented for STIX image reconstruction. These algorithms are selected for their distinctive features, which will be discussed in this section. Below is a brief description of each algorithm.

*VIS_FWDFIT*: is based on the assumption that the sought image consists of a superimposition of parametric shapes, e.g., bidimensional Gaussian functions [28]. Once the user has selected the number and type of shapes to describe the image to be reconstructed, the algorithm fits the parameter values from the observed visibilities by solving an optimization problem. In the STIX implementation, this optimization problem is solved using the particle swarm optimization algorithm [29].
*MEM_GE*: retrieves the image with the highest entropy among those that fit the data with the same level of accuracy [13]. The method also constrains the pixel values to be non-negative and ensures that the sum of the pixel values equals an a priori estimate of the total X-ray emission.
*CLEAN*: was developed in the mid-1970s to address the challenge of synthesizing high-resolution images from Fourier data collected by radio telescopes [30]. Its development was pivotal for radio astronomy, enabling clearer and more detailed images of celestial objects. CLEAN is a deconvolution algorithm, which iteratively removes the contribution of point sources from the dirty image, i.e., the convolution of the underlying (and unknown) X-ray emission with the instrument point spread function. At the end of the iterative process, the CLEAN components image, which consists of the identified point sources, is convolved with a Gaussian beam to get the final output of the method.

We summarize the algorithms based on their *processing approach* and *input data types*, and list their *time-to-solution* data in seconds for each reconstructed image in a 1000-sample dataset^3^ in Table 1.

**Table 1:** Summary of STIX image reconstruction algorithms, including processing approach, input data, and time-to-solution

Imaging Algorithm	Processing Approach	Input Data Type	Time-to-Solution (s)
VIS_FWDFIT	Parameter-dependent	Visibilities	5.522 ± 3.655
MEM_GE	End-to-end	Visibilities	9.048 ± 6.844
CLEAN	End-to-end	Dirty Image	0.032 ± 0.092

While MEM_GE and CLEAN are end-to-end algorithms, VIS_FWDFIT is parameter-dependent in terms of processing approach, as summarized in Table 1. In other words, MEM_GE and CLEAN can be run directly with the given input, providing the output to the user without requiring manual intervention. In contrast, VIS_FWDFIT requires the input of additional parameters from a human expert. Table 1 also highlights the variability in computation times for each reconstruction algorithm. While CLEAN is relatively fast compared to the other two, VIS_FWDFIT and MEM_GE take approximately 5 and 9 s, respectively, for each reconstructed image.

**Fig. 2:**
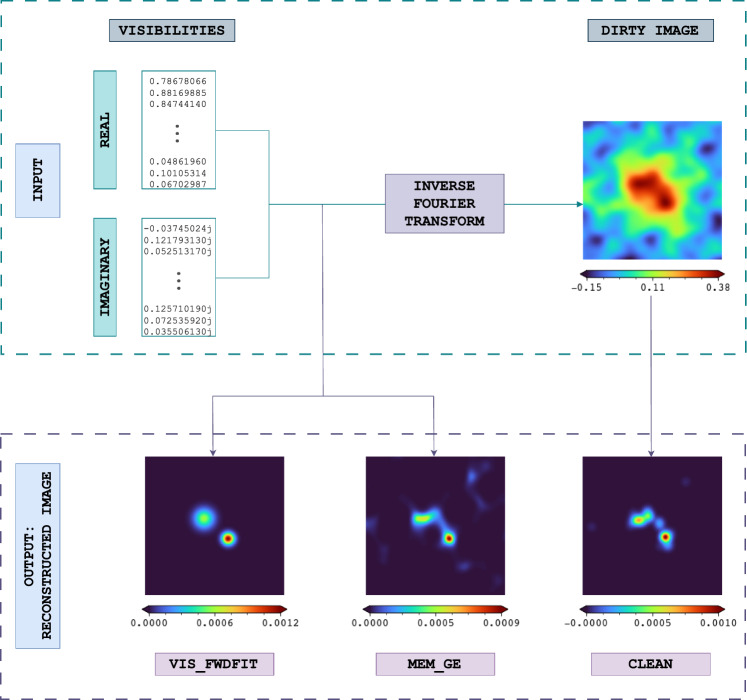
Process of image reconstruction algorithms applied to STIX data

Figure 2 illustrates the overall imaging process in STIX. The visibilities, consisting of both real and imaginary parts of the Fourier components, are either fed directly into the algorithms or converted into a dirty image through a direct Fourier inversion. Table 1 lists, while Fig. 2 depicts, the input data type for each STIX image reconstruction algorithm. The reconstructed images, plotted as color maps in Fig. 2, represent the intensity of the X-ray radiation that is emitted from the Sun and is measured by STIX. Note that due to the non-uniqueness of the solution for STIX imaging, different imaging techniques yield slightly varying, yet consistent, results, as can be observed in the *output* part of Fig. 2. Further, the reconstructed STIX images can contain artifacts, i.e., unreal areas of intense emission created by the reconstruction method and due to the limited number of available visibilities (see, e.g., the bright feature in the top-right corner of the MEM_GE reconstruction in Fig. 2). Given the variable runtime of the implemented image reconstruction algorithms, as shown in Table 1, along with the differences in their outputs and the presence of artifacts in the reconstructions, we propose that solar image reconstruction could benefit from a deep learning approach.

### Data acquisition, preparation, and processing: observational and simulated STIX Data

In this study, we utilized both observational and simulated STIX data to develop and validate our image reconstruction approach. The observational data were obtained directly from STIX observations of solar flares [17] and were used solely in the testing phase of our approach. Since the ground truth image for an actual STIX observation is unknown and therefore unavailable for training the FCD, we generated simulated STIX data both to train the network and to complement the observational data in testing.

#### Simulation methodology

The simulation methodology involves generating synthetic STIX data designed to approximate actual solar flare observations while allowing for controlled experimentation. Due to the instrument resolution, the limited number of visibilities measured by STIX, and the underlying physics of solar flares, the reconstructed images of the flaring X-ray emission can be approximated as a superimposition of 2D Gaussian shapes [28, 31]. The data simulator we implemented takes only the number of X-ray sources as input and generates a random configuration of Gaussian functions, along with the corresponding visibilities that would have been measured by STIX.

A single Gaussian source is defined by six parameters: the *x* and *y* coordinates of the center, the minimum and maximum full width at half maximum (FWHM), the orientation angle of the Gaussian, and the total intensity, i.e., the integral of the function [31]. To simulate a configuration with a single source, our code randomly draws the values of these six parameters from appropriate distributions. It then applies Equation (35) from [24] to analytically compute the number of counts that would be measured by the STIX detectors if such a Gaussian X-ray source were observed.

Next, the simulator perturbs the number of counts with Poisson noise and applies Equations (18) and (19) from [24] to derive the visibility values that would be measured by each of the 24 STIX sub–collimators. If more than one source is simulated, these operations are repeated for each Gaussian function in the configuration, and the corresponding visibilities are summed together.^4^ The outputs of the simulator include the configuration parameter values and the corresponding visibilities. From the parameter values, the ground truth image of the Gaussian configuration used to train the FCD can be constructed.

**Fig. 3:**
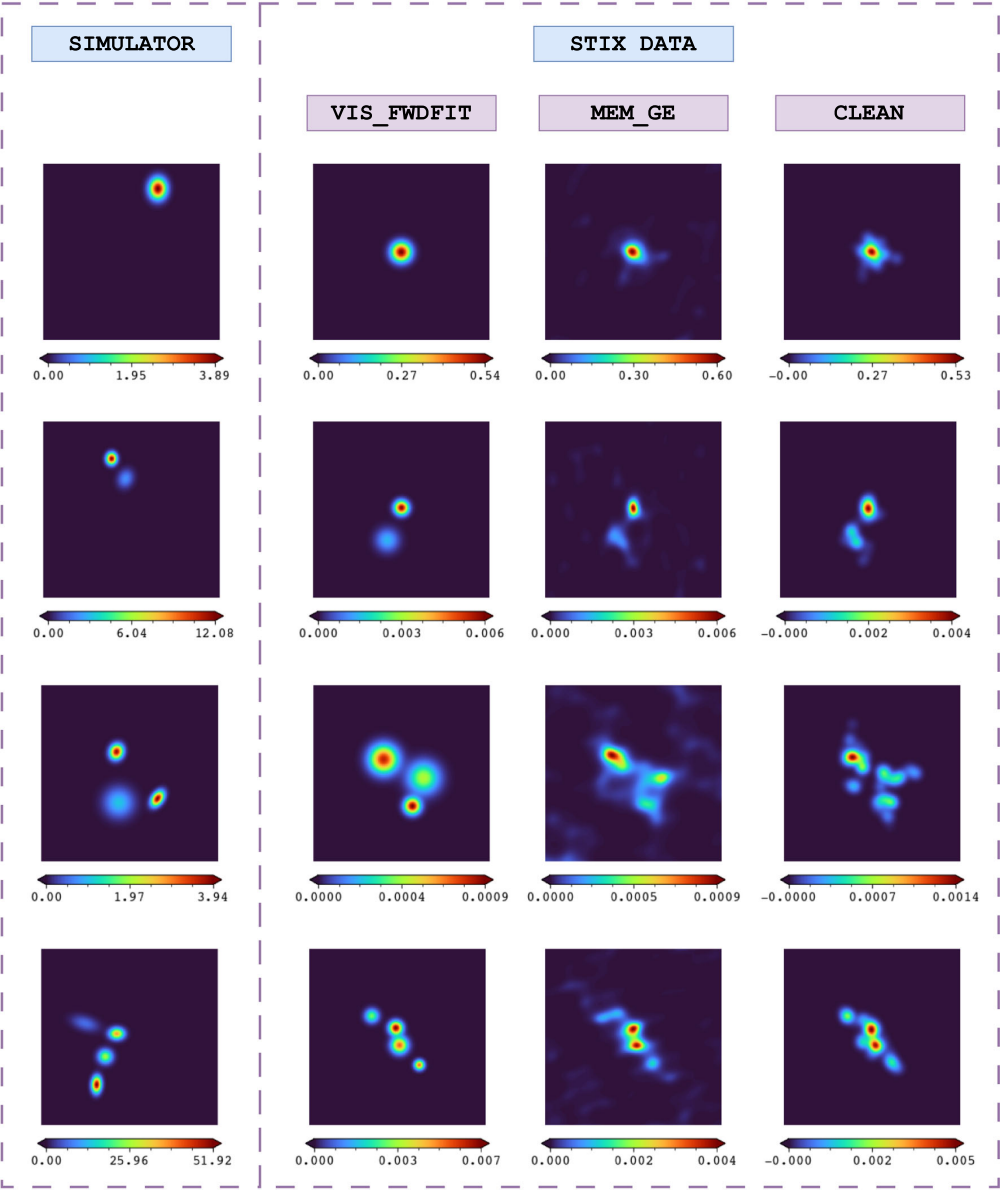
Comparison of simulated images and reconstructed images derived from STIX data by source count

The number of X-ray sources in solar flares varies, but it is usually very low, typically one or two. However, there are instances where a larger number of sources is observed, such as in the event studied in [32], where X-rays were emitted from four distinct locations. Although flares with more than two sources are rare, these cases are included in our simulated dataset to ensure that the network has an equitable opportunity to recognize and reconstruct data across all source configurations. Consequently, our dataset contains simulated data corresponding to configurations with one to four sources. Figure 3 illustrates and compares the simulated images and reconstructed images derived from observational data based on the number of sources.

#### Data normalization in simulated data

The simulated data are normalized for both training and testing purposes. Normalization of the simulated data is of significant importance to ensure that the deep learning model can effectively learn from it. Additionally, in solar flare image reconstruction, where accurate representation of flux densities is crucial, normalizing the data was a key step to ensure consistency and reliable model performance.

The total intensity of the simulated configurations is randomly drawn from a power law distribution, ranging from approximately 1000 to 500,000 photon counts per second ($\hbox {s}^{-1}$), per square centimeter ($\hbox {cm}^{-2}$), per kilo-electronvolt ($\hbox {keV}^{-1}$), with a power law index of 2. As a result, because the Fourier transform is a linear operator, the visibility values of the simulated data points span more than two orders of magnitude. To reduce the range of the FCD input values and eliminate dependency on the total flux value (which is merely a scaling factor), we normalized each simulated visibility set and the corresponding ground truth image as follows. The visibility amplitude is defined as $\vert V \vert = \sqrt{\Re V^2 + \Im V^2}$, where $\Re V$ represents the real and $\Im V$ the imaginary parts of the visibility components. For each simulated sample, we compute:

1 $$\begin{aligned} \alpha = \frac{1}{2} \times \max _{i=1,\dots,24} \vert V_i \vert ~, \end{aligned}$$

where the factor 1/2 is chosen to maintain a consistent input range for the network. We opted for the range [$-$2,2] instead of more conventional [$1,1$] to ensure that the network can effectively capture the dynamic range of the input data without saturation or loss of information. Both the real and imaginary parts of the visibilities and the ground truth image are divided by $\alpha$. This normalization process ensures that the visibility values provided as input to the network are within the range [$-$2,2], and the sum of the pixel values of the ground truth images is approximately 1/2. Simulated data values before and after applying the $\alpha$ coefficient are presented in Table 2.

**Table 2:** Numerical data values for visibilities and ground truth images before and after normalization, including mean values and corresponding standard deviations. Some values are rounded to two decimal places, rounding up if the third digit is 5 or above

	Pre Normalization		Post Normalization	
	Visibilities	Images	Visibilities	Images
Mean	520.51 ± 2837.47	0.08 ± 0.71	0.2 ± 0.83	3.07e$-$05 ± 0.0002
Minimum	$-$34513.25	0.0	$-$2.0	0.0
Maximum	38953.73	101.19	2.0	0.02

The normalization allows the FCD to process numbers within a consistent range, independent of the original visibility scale. Moreover, during inference, the actual total intensity of the reconstructed image can be retrieved by multiplying the FCD output by the same $\alpha$ value used to normalize the corresponding input visibilities.

#### Observational data handling

Automatically constructing a dataset of STIX images from raw observations presents a challenging task. Reconstructing a STIX image requires integrating raw detector measurements over specific time and energy intervals, ideally necessitating an automatic procedure to define the optimal ranges for each flaring event. The imaging products currently held in the STIX data center are the result of over 50,000^5^ flaring events, constructed using the procedure described in [17]. However, our data analysis reveals that the procedure has not been fully optimized, leading to many reconstructions that are unsuitable for scientific analysis.

In the following, we summarize the steps implemented to remove scientifically insignificant images. First, we discarded all images reconstructed from data with fewer than 5000 total photon counts, as the average noise level affecting the STIX measurements at this threshold is approximately 20%.^6^ Next, we removed images with an integration time exceeding 5 min.^7^ Finally, we discarded images that were corrupted due to factors such as misestimated flare location or incorrect instrument pointing information. At the end of the cleaning process, the final dataset consists of 4274 flaring events.

#### Final data preparation

Both of our datasets consist of visibility vectors, sized 48$\times$1, to be used as input, and the corresponding reconstructed images, sized 128$\times$128, to be used as output. All visibility vectors initially comprise real and imaginary Fourier components, each with a default dimension of 24$\times$1. The components are then sequentially combined, resulting in a 48$\times$1 vector, and are stored as real numbers before further processing.

While the simulated dataset includes visibility vectors, corresponding ground truth images, and the $\alpha$ coefficients elaborated in Sect. 2.3.2, the observational dataset includes visibility vectors and the corresponding reconstructed images by VIS_FWDFIT, MEM_GE, and CLEAN. Note that CLEAN images were initially reconstructed as 129$\times$129 by IDL, the software used for CLEAN, which reconstructs CLEAN images with odd dimensions. Such images were then resized to 128$\times$128 using area-based resampling in order to maintain consistency with the other datasets.

## Approach: a custom-made and supervised autoencoder model

In this research, we opted for a specialized, supervised autoencoder as the artificial neural network architecture. An autoencoder typically consists of two main components: an encoder and a decoder. In a common scenario, the encoder compresses the input into a lower-dimensional latent space, and the decoder reconstructs the input from this latent representation [33]. Although autoencoders often aim to have the input and output data share the same dimensions, this is not always required. The reconstruction process can be either deterministic, as in standard autoencoders, or probabilistic, as in variational autoencoders [4].

What differentiates our approach is that our input data, i.e., 1D vectors, have lower dimensions than the output data, i.e., 2D images. Consequently, our autoencoder is overcomplete, meaning the latent space has a larger dimension than the input. A variational output is not suitable for our task, as we focus on image reconstruction rather than image generation. Therefore, the latent space in our autoencoder is deterministic, ensuring that the encoder consistently produces the same decoded representation for a given input, making it well-suited to our needs. Finally, our autoencoder is trained in a supervised manner to approximate a ground truth image from the corresponding simulated Fourier components, leveraging the deterministic latent space to ensure consistent and accurate reconstructions.

The Fourier components, consisting of both real and imaginary parts, are merged into a single vector by concatenating the real and imaginary components sequentially. This combined vector is fed into the encoder, resulting in a dense representation of the vectors in the latent space. This representation is then passed to the decoder, producing a reconstructed image.

### Neural network architecture

We employ a multilayer perceptron (MLP) as the encoder, taking advantage of its ability to learn and approximate complex functions, particularly when applied to our input data, which consists of Fourier components, i.e., visibilities. To mitigate the risk of overfitting, which is common in MLPs, we utilize normalization layers and implement an early stopping mechanism during training.

The decoder consists of a series of up-convolution layers. Each set includes an up-sampling layer, followed by a convolutional layer, a normalization layer, and concludes with a learnable activation function applied on top. Up-sampling layers increase the spatial dimensions of the feature maps, which in this setup is achieved through nearest neighbor interpolation by repeating the rows and columns of the data. The convolutional layers then refine these up-sampled feature maps to produce a more accurate reconstruction.

Before settling on up-convolution layers, we experimented with deconvolutional (or transposed convolutional) layers [34, 35] as an alternative. Both types of layers are used to increase the spatial dimensions of feature maps but are implemented differently. Our experiments revealed that deconvolutional layers introduce artifacts in the output images, such as checkerboard or mosaic patterns, which are known side effects of deconvolution operations [36, 37]. Consequently, the final version of the decoder incorporates up-convolution layers to reconstruct the images without artifacts.

Given that the network uses Fourier components as inputs and convolutional layers in the decoder to determine the output, we find the name Fourier convolutional decoder (FCD) appropriate for our architecture.

**Fig. 4:**
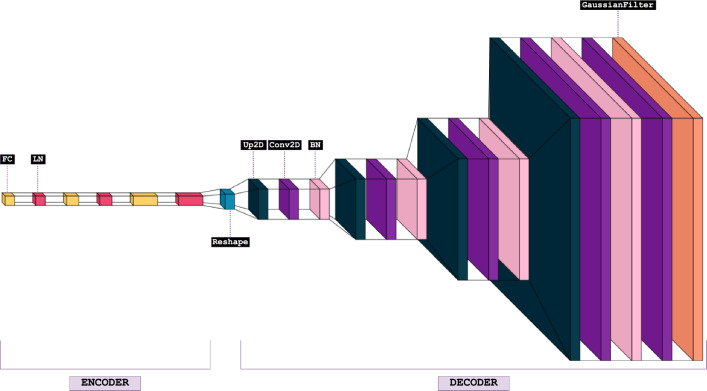
Architecture of the FCD, highlighting layer names and the division between encoder and decoder components$^8$

The architecture of the FCD is displayed in Fig. 4. The following acronyms are used in the figure, with their corresponding explanations:

- FC: Fully connected (densely connected) layer.
- LN: Layer normalization.
- UP2D: Up-sampling for 2D data.
- CONV2D: Convolution for 2D data.
- BN: Batch normalization.

8

The encoder in Fig. 4 comprises three repetitive sets of fully connected layers followed by layer normalization, continuing up to the *reshape* layer. The input to the encoder is a 48$\times$1 vector, formed by concatenating the 24 real and 24 imaginary components of the Fourier data, corresponding to 24 visibilities, into a single vector. The decoder begins with the reshaped input from the encoder and consists of four up-convolution groups. The final two layers of the decoder include a convolutional layer and a Gaussian filter, ensuring a smooth output, with the final output being a 128$\times$128 image. The FCD contains 961,089 parameters. The number of neural units in both the encoder and the decoder was determined through hyper-parameter tuning, with a consistent kernel size of 5$\times$5 across the FCD. Details of the FCD layers and corresponding output shapes, including sizes, are provided in Table 3. The remaining aspects of the FCD architecture are discussed in the following sections.

**Table 3:** Summary of FCD layers and corresponding output shapes

	Layer Type	Output Shape
Encoder	FC	128
	PReLU + LN	128
	FC	256
	PReLU + LN	256
	FC	512
	PReLU + LN	512
	Reshape	8$\times$8$\times$8
Decoder	Up2D	16$\times$16$\times$8
	Conv2D	16$\times$16$\times$128
	BN + PReLU	16$\times$16$\times$128
	Up2D	32$\times$32$\times$128
	Conv2D	32$\times$32$\times$64
	BN + PReLU	32$\times$32$\times$64
	Up2D	64$\times$64$\times$64
	Conv2D	64$\times$64$\times$32
	BN + PReLU	64$\times$64$\times$32
	Up2D	128$\times$128$\times$32
	Conv2D	128$\times$128$\times$16
	BN + PReLU	128$\times$128$\times$16
	Conv2D (+Sigmoid)	128$\times$128$\times$1
	Gaussian Filter	128$\times$128$\times$1

#### Activation function

Parametric rectified linear unit (PReLU) [39] is utilized in both the encoder and decoder sections of the FCD. Given that the visibility vectors include both negative and positive floating point values,^9^ PReLU was selected to preserve these values and enhance the FCD’s ability to learn effectively. As an adaptable activation function, PReLU integrates into the FCD’s learning process, making it well-suited for handling diverse input characteristics. Our hyper-parameter tuning experiments, which included evaluating various activation functions such as Gaussian error linear unit (GeLU) [40], confirmed that PReLU was the most effective choice. Unlike GeLU, which processes negative values in a smoother, probabilistic manner, PReLU offers flexibility in handling negative values through a learnable parameter. This flexibility allows PReLU to better capture the nuanced variations in the input data, enhancing the FCD’s learning capacity. Additionally, the activation function in the final convolutional layer, immediately before the output, is Sigmoid, which ensures that the output values are appropriately scaled and confined to a specific range.

#### Normalization layers

Normalization layers are employed throughout the FCD to stabilize and accelerate the training process. Layer normalization (LN) [41] is applied after every fully connected layer in the encoder, while batch normalization (BN) [42] with a batch size of 128 is used after each up-convolutional layer set in the decoder. BN operates on individual batches, normalizing across the batch dimension, whereas LN normalizes across the entire feature dimension. Given our input data, which spans both negative and positive values, we selected LN for the encoder to enhance the FCD’s generalization capabilities. This decision was further supported by hyper-parameter tuning experiments. In the decoder, the spatial structure of the convolutional layers benefits from normalization across the batch dimension, making BN the preferred choice for promoting the learning of robust feature maps.

#### Gaussian filtering

The STIX images consist of features that can be well-approximated by Gaussian functions and shapes with soft edges, as previously shown in Fig. 2. Therefore, it is necessary that the FCD provides smooth output results. To reduce artifacts generated by the FCD, we switched from deconvolutional layers to up-convolution layers and integrated a Gaussian filtering layer into the model after the final convolutional layer. This addition helps further eliminate noise and smooth the predictions produced by the FCD. The Gaussian filter used is isotropic, meaning it smooths equally in all directions, which is advantageous for preserving circular or spherical features. Additionally, it effectively reduces noise without causing significant distortion [43]. The Gaussian filtering layer incorporated into the FCD is nonparametric, meaning it does not have learnable weights. The adopted 2D Gaussian filter function is

2 $$\begin{aligned} G(x, y) = \frac{1}{2\pi \sigma ^2} e^{-\frac{x^2 + y^2}{2\sigma ^2}} ~, \end{aligned}$$

where (*x*, *y*) indicates the pixel location in a 2D space, and sigma ($\sigma$) determines the smoothness coefficient. The Gaussian filtering layer in the FCD is implemented using a depth-wise separable 2D convolutional layer with a kernel size of 5$\times$5 and a $\sigma$ value of 0.7. In this context, $\sigma$ controls the width and represents the standard deviation of the Gaussian distribution, while the kernel size determines the number of pixels considered during filtering.

After incorporating up-convolution and Gaussian filtering layers into the FCD architecture, we evaluated the smoothness achieved at each step by measuring the Laplacian variance [44]. Laplacian variance is a metric used to assess the smoothness or sharpness of an image [45]. In the context of image processing, smoothness corresponds to minimal changes in intensity, while sharpness indicates the presence of edges and fine details. The Laplacian operator, a second-order derivative, detects rapid changes in intensity, effectively highlighting edges. The variance of these Laplacian values, known as Laplacian variance, quantifies the smoothness of the image. A high Laplacian variance indicates significant intensity variation, suggesting a sharper image, whereas a low variance corresponds to fewer edges, indicating a smoother image.

**Fig. 5:**
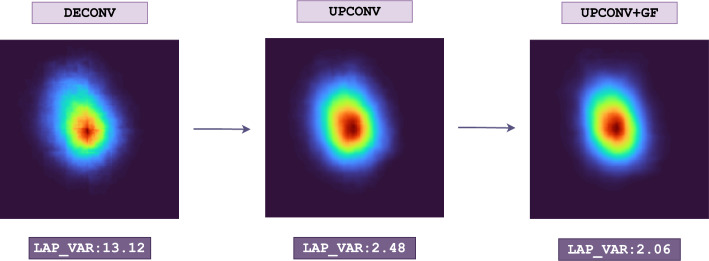
Three stages in the smoothness experiments, along with the corresponding Laplacian variance results

Figure 5 illustrates the stages of the smoothness experiments we conducted, together with the corresponding decreases in Laplacian variance at each stage. The first (1^st^) stage represents the *deconvolution* process on the left, the second (2^nd^) stage involves *up-convolution* in the middle, and the third (3^rd^) stage on the right combines *up-convolution and Gaussian filtering* at the end. There is an approximate 81.1% increase in smoothness between the 1^st^ and 2^nd^ stages, with an additional approximate 16. 9% increase between the 2^nd^ and 3^rd^ stages. Overall, the Laplacian variance decreases by approximately 83.5% between the 1^st^ and 3^rd^ stages for the sample shown in Fig. 5. The experiments exploring different kernel sizes and $\sigma$ values revealed that the selected parameters strike a balance between achieving smoothness and avoiding the blurring of fine details, which finalized the FCD architecture.

### Model training details

*Training and Validation Data*: A total of 200,000 training samples, along with 40,000 validation samples, were used during the training process. The dataset was shuffled before training, and the entire training set consists of simulated data.
*Optimization Method*: We employed the Adam optimizer [46], an adaptive optimization algorithm known for its effectiveness in handling gradients of varying scales.
*Custom Loss Function*: A combined loss function incorporating mean absolute error (MAE) and binary cross-entropy (BCE) is used to balance two objectives: achieving well-defined shapes through BCE and accurately reconstructing images that reflect the ground truth through MAE. One challenge with using this combined loss function, as observed in our case with our data, is its sensitivity to small values, which can result in gradients that are too small to drive effective learning. To address this, custom loss scaling was applied by multiplying the combined loss by 1000, ensuring that the gradients are large enough to provide meaningful updates during training. The adopted loss function is then
  3 $$\begin{aligned} L(I_{\text{GT}}, I_{\text{rec}}) = 1000 \cdot \left( \text{MAE}(I_{\text{GT}},I_{\text{rec}}) + \text{BCE}(I_{\text{GT}}, I_{\text{rec}}) \right) ~, \end{aligned}$$
  where $I_{\text{GT}}$ is the ground truth image, $I_{\text{rec}}$ is the image reconstructed by the model,
  4 $$\begin{aligned} \begin{aligned} \text{MAE}(I_{\text{GT}}, I_{\text{rec}})&= \frac{1}{P} \sum _{i=1}^P \vert (I_{\text{GT}})_i - (I_{\text{rec}})_i \vert ~,\\ \text{BCE}(I_{\text{GT}}, I_{\text{rec}})&= -\frac{1}{P} \sum _{i=1}^P ((I_{\text{GT}})_i \cdot \log ((I_{\text{rec}})_i)+(1-(I_{\text{GT}})_i)\cdot \log (1-(I_{\text{rec}})_i )) ~, \end{aligned} \end{aligned}$$
  and $P = p \times p$ denotes the number of pixels in the images. The choice of MAE over the more commonly used mean squared error (MSE) in image generation and reconstruction tasks was driven by the nature of our image data, which contains low floating point values as pixel intensities. This choice helps in better capturing these small variations in the data.
*Learning Rate Scheduling*: Optimal utilization of the learning rate involves progressively reducing it throughout the training process. This reduction can be achieved either internally, by assigning a steady decay rate and predefined steps, or externally, by monitoring the behavior of the loss function and decreasing the learning rate when the loss stagnates. Our experiments demonstrated that FCD benefited more from a controlled internal reduction, resulting in improved generalization in the outputs. This technique, known as a dynamic learning rate, gradually reduces the rate as the training progresses. Employing a dynamic learning rate allows the model to steadily approach convergence, minimizing the error. The initial learning rate was set at 0.001, and gradually decreased to 0.0009372 at the end of the training process. We assigned a decay rate of 1 and set the decay steps at * (training set size / batch size)* $\times$ 1000.
*Early stopping & Number of epochs*: An early stopping mechanism was implemented during training to prevent overfitting and to cease training once convergence was achieved. The validation loss was monitored for early stopping, with a patience of 10 epochs. The final model was selected based on the epoch that yielded the best weights. The current version of the model required 66 epochs to train, with a batch size of 128, taking approximately 2.5 h.
*Hardware specifications*: The FCD was trained on an NVIDIA RTX A4500 GPU.

## Algorithmic framework for visibility-based image reconstruction

**Fig. 6:**
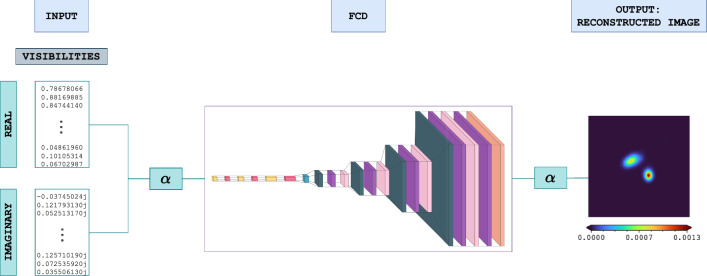
Complete framework of the proposed system$^{10}$.

This section presents a step-by-step description of the image reconstruction process using our approach. It begins with processing the measured visibilities, followed by passing them through our custom-designed autoencoder, the FCD, and ends with obtaining the reconstructed image. The complete framework is shown in Fig. 6, which provides a high-level overview of the workflow. The corresponding algorithm is outlined in Algorithm 1.

10

In the reminder of the paper, we will denote:

- $V_j$: the *j*-th visibility measured by the instrument, where $j=1,\dots,N$ and $N=24$ is the total number of visibilities recorded by STIX;
- $\mathcal {V} = (V_1, \dots, V_N)$: the complex array of measured visibilities;
- $\textbf{V} = (\Re \mathcal {V}, \Im \mathcal {V})$: the array of real numbers formed by concatenating the real and imaginary parts of $\mathcal {V}$.

**Algorithm 1:**
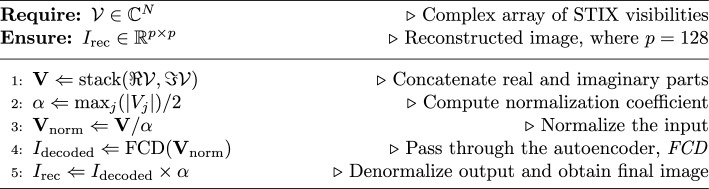
Visibility-based image reconstruction using FCD

The reconstruction pipeline follows these steps, which are illustrated in Fig. 6 and technically described in the pseudo-code (Algorithm 1):

1. *Input Preparation* The visibilities are stacked by concatenating the real and imaginary parts, producing a combined input array $\textbf{V}$.
2. *Normalization* The input array is normalized using a coefficient $\alpha$, computed as half the maximum amplitude of the visibility values. This normalization step ensures that the data are scaled appropriately for the autoencoder.
3. *Autoencoder Processing* The normalized visibility data are passed through the autoencoder. First, the data are encoded by the encoder, and then the decoder reconstructs the image based on the encoded features.
4. *Denormalization* The output from the autoencoder is denormalized by multiplying by the normalization coefficient $\alpha$ to recover the scale of the original data.
5. *Final Output* The final reconstructed image is obtained after denormalization, representing the flaring X-ray emission.

The method follows a structured pipeline, with each step listed in Table 4, which aligns the algorithmic steps with the corresponding processing steps in the workflow.

**Table 4:** Mapping of processing steps to algorithm steps

Processing Step	Algorithm Step
Input (Fourier components)	Step 1
Normalization using $\alpha$ coefficient	Steps 2–3
FCD (Autoencoder)	Step 4
Denormalization and final output (Reconstructed image)	Step 5

## Evaluation metrics

### Image reconstruction metrics

We use a set of five metrics to evaluate the image reconstruction quality by comparing the ground truth image from the simulated data with the image reconstructed by the FCD. Among these, the Dice similarity coefficient and Hausdorff distance are applied to binary images; multi-scale structural similarity index measure (SSIM) and peak signal-to-noise ratio (PSNR) are used on single-channel (grayscale) images; and learned perceptual image patch similarity (LPIPS) is applied to three-channel (colored) images. Since our data consist of single-channel images, we first convert them into the appropriate format for each metric. The details of these conversions, as well as the rationale for selecting this set of metric, are discussed throughout this section.

#### Dice similarity coefficient

The Dice coefficient, also known as the Sørensen-Dice coefficient, is commonly used in image segmentation tasks and is equivalent to the *F1* score in the context of binary classification. Therefore, the Dice coefficient can be considered a specific case of the F1 score when evaluating the overlap between two sets or the accuracy of classifications. The Dice coefficient ranges from 0 to 1, with 1 indicating perfect similarity and 0 indicating no similarity.

**Fig. 7:**
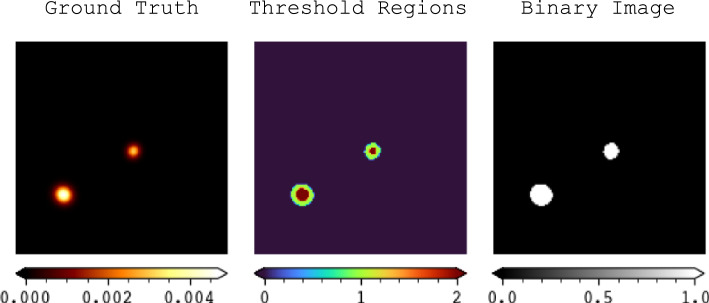
Application process of binarization on test sample 18347/40000

Binarization in image processing is achieved through thresholding. Figure 7 illustrates the process of binarizing our data. In the figure, *Ground Truth* refers to the simulated image containing two X-ray sources. *Threshold Regions* display the multiple regions in the test image obtained using the fast multi-Otsu thresholding algorithm [47]. *Binary Image* shows the result after applying Otsu’s algorithm.

In Eq. (5), *DSC* represents the dice similarity coefficient, which is calculated as 2 times the intersection over the union of two binarized images, X and Y:

5 $$\begin{aligned} DSC = \frac{2 |X \cap Y|}{|X| + |Y|} \end{aligned}$$

#### Multi-scale structural similarity index measure (MS-SSIM)

SSIM focuses on luminance, contrast, and structural information to evaluate perceptual quality [48]. The maintenance of uniform regions and the preservation of shapes are crucial to achieve a high SSIM score, making it valuable for applications like ours that require geometric precision and clarity.

Given that we work with scientific images whose pixel values vary significantly, as shown earlier in Table 2, we opted for the multi-scale (MS) version of SSIM [49] as an image reconstruction evaluation metric. MS-SSIM results range from 0 to 1, with higher values indicating greater similarity between the images.

6 $$\begin{aligned} \text {MS-SSIM}(x, y) = \left[ l_M(x, y) \right] ^{\alpha _M} \cdot \prod _{j=1}^{M} \left[ c_j(x, y) \right] ^{\beta _j} \left[ s_j(x, y) \right] ^{\gamma _j} \end{aligned}$$

The parameters of MS-SSIM presented in Eq. (6) are explained below:

- *M*: The number of total scales in a multi-scale environment.
- $l_M(x, y)$: The luminance comparison function at the *M*-th (i.e., highest) scale.
- *j*: The index of *M*.
- $c_j(x, y)$: The contrast comparison function at scale *j*.
- $s_j(x, y)$: The structure comparison function at scale *j*.
- $\alpha _M$, $\beta _j$, and $\gamma _j$: The weights assigned to the luminance, contrast, and structure components, respectively.

Details of the *luminance*, *contrast*, and *structure* functions can be found in [49].

#### Perceptual image quality: LPIPS

LPIPS leverages deep learning to assess perceptual similarity between images, making it more aligned with human visual perception [50]. Given the importance of human perception in astrophysical image reconstruction, we report LPIPS values alongside other metrics. LPIPS utilizes a pre-trained deep neural network-*AlexNet* in our case-to extract high-level features from image patches. The perceptual distance is then computed as:

7 $$\begin{aligned} \text {LPIPS}(x, y) = \sum _\text {l} w_\text {l} \Vert f_\text {l}(x) - f_\text {l}(y) \Vert _2^2 \end{aligned}$$

where $f_l$ represents the features extracted at layer *l* of the network, and $w_l$ are learned weights. The output range of LPIPS is from 0 to 1, with 0 indicating perfect perceptual similarity. Note that LPIPS requires three-channel (RGB) images, while we have single-channel (grayscale) images. To address this, we convert the 2D grayscale array into a 3-channel RGB array, where each channel (R, G, B) contains the same values.

#### Peak signal-to-noise ratio (PSNR)

PSNR is a metric used to assess the quality of a reconstructed image by comparing it to the ground truth. Because it is sensitive to pixel differences, PSNR is effective in identifying and quantifying image degradation. The formula for calculating PSNR is provided in Eq. (8). In this equation, MAX represents the maximum pixel value of the ground truth image, and MSE denotes the mean squared error between the ground truth and the reconstructed image generated by the FCD. A higher PSNR value indicates a higher quality of the reconstructed image.

8 $$\begin{aligned} \text {PSNR} = 10 \cdot \log _{10} \left( \frac{\text {MAX}^2}{\text {MSE}} \right) \end{aligned}$$

The PSNR measures the ratio between the peak signal level and the background noise, making it a useful metric for assessing image noise. In this context, a higher PSNR value corresponds to a lower noise level. Beyond evaluating the quality of reconstructed images, we also use PSNR to quantify the noise level in the simulated environment, where the ground truth is available.

#### Hausdorff distance

The Hausdorff distance is a metric that measures the maximum distance between points in two sets and is commonly used to determine the similarity between two shapes or sets of points.

The Hausdorff distance $d_H$ between two non-empty subsets $A$ and $B$ is defined as:

9 $$\begin{aligned} d_H(A, B) = \max \left\{ \sup _{a \in A} \inf _{b \in B} d(a, b), \sup _{b \in B} \inf _{a \in A} d(b, a) \right\} \end{aligned}$$

where $d(a, b)$ represents the distance between the points $a$ and $b$ in the metric space. The distance $d_H(A, B)$ considers the maximum of *h*(*A*, *B*) and *h*(*B*, *A*) to ensure symmetry [51]. A smaller Hausdorff distance indicates a greater similarity between the two sets.

### Data reconstruction metrics

The second set of metrics we use is designed to evaluate the ability of the imaging methods to reconstruct images that are consistent with the input data. Here, input “data” refers to the visibilities measured by STIX.

In this section, we denote $\hat{V}_j$ the *j*-th visibility predicted from a reconstructed image, which is obtained by applying the Fourier operator, i.e., by computing

10 $$\begin{aligned} \hat{V}_j = \iint I(x,y) \exp (2\pi i (xu_j+yv_j)) \,dx\,dy ~,~ j=1,\dots,N \end{aligned}$$

where *I*(*x*, *y*) denotes the intensity of the reconstructed image at pixel (*x*, *y*) and $(u_j,v_j)$ represents the *j*-th frequency sampled by STIX. Note that, in practice, the integral of Eq. (10) is replaced with a discrete approximation. Finally, we denote by $\hat{V}$ the complex array containing the visibilities predicted from a reconstructed image and by $\hat{\textbf{V}} = (\Re \hat{V}, \Im \hat{V})$ the concatenation of the real and the imaginary parts of $\hat{V}$.

#### Chi-squared metric

The chi-squared (${\chi }^2$) metric evaluates the statistical consistency between the visibility values predicted from the reconstructed image, using Eq. (10), and the observed visibilities provided as input to the FCD by STIX. This metric, which is widely adopted for assessing the reliability of images reconstructed from X-ray visibilities [13, 52], is defined as

11 $$\begin{aligned} \chi ^2 = \frac{1}{N-1} \sum _{j=1}^N \frac{\vert V_j - \hat{V}_j \vert ^2}{\sigma _j^2} ~, \end{aligned}$$

where $\vert \cdot \vert$ represents the visibility amplitude defined earlier in Eq. (1), and $\sigma _j$ is the statistical uncertainty^11^ on the corresponding visibility amplitude.

#### Mean absolute error

We use the MAE metric to measure the differences between the magnitudes of the vectors. Equation (12) calculates the error magnitude by computing the average of the absolute differences between the true and predicted vectors, as shown below:

12 $$\begin{aligned} \text {MAE} = \frac{1}{2N} \sum _{j=1}^N |\Re V_j - \Re \hat{V}_j| + |\Im V_j - \Im \hat{V}_j| \end{aligned}$$

#### Cosine similarity

Cosine similarity measures the cosine of the angle between two nonzero vectors in an inner product space. We use the *cosine similarity* metric to assess the phase similarity, or alignment, between two vectors. Equation (13) calculates the cosine of the angle between the vectors in their multidimensional space, capturing the similarity in direction between them, regardless of their magnitude. A higher cosine similarity value indicates greater similarity between the two vectors.

13 $$\begin{aligned} \text {Cosine Similarity} = \frac{\textbf{V} \cdot \hat{\textbf{V}}}{\Vert \textbf{V}\Vert \Vert \hat{\textbf{V}}\Vert } \end{aligned}$$

#### Spectral convergence

Finally, we use the *spectral convergence* metric to measure how closely the predicted spectrum converges to the actual spectrum. In the context of signal processing, *spectral convergence* is commonly used to assess the difference between the spectra of two successive frames, serving as an indicator of the local smoothness of a spectrogram. A lower spectral convergence value suggests that the spectral content of the signal changes slowly over time, which is often desirable as it indicates a more stable or consistent signal. Conversely, a higher value indicates rapid changes in the spectral content. Therefore, a lower spectral convergence value reflects a better result for this metric, which is defined as

14 $$\begin{aligned} \text {Spectral Convergence} = \frac{\Vert \textbf{V} - \hat{\textbf{V}} \Vert }{\Vert \textbf{V} \Vert } \end{aligned}$$

In Eq. (14), *Spectral Convergence* is defined as the ratio of the norm of the difference between the predicted and input visibilities to the norm of the input visibilities.

## Results and discussion

We conducted experiments on two different datasets and evaluated them using metrics tailored to each specific case. Table 5 presents a summary of the overall data used in this research. The table shows that there are two sets of simulated data. The larger set, comprising 280,000 samples, is used for both training and testing the FCD. A subset of the simulated data, consisting of 1000 samples, is exclusively used for testing, which also involves the STIX image reconstruction algorithms MEM_GE, VIS_FWDFIT, and CLEAN.

**Table 5:** Numerical summary of datasets utilized in this research

	Data Count				
Dataset	Total	Train	Test	Validation	Dataset Size
Simulated Data	280,000	200,000	40,000	40,000	17.14 GiB
Simulated Data - *Subset*	1000	–	1000	–	62.69 MiB
Observational (STIX) Data	4274	–	4274	–	1.57 GiB

As outlined earlier in Sect. 2.2, the CLEAN imaging algorithm produces a map composed of point sources (clean components), which is then convolved with a Gaussian beam. For the data reconstruction experiments, the clean components map is used, while the convolved map is applied in the image reconstruction experiments.

Note that throughout this section, all metric results in the tables are presented as mean values with corresponding standard deviations. Values are rounded to two decimal places, rounding up if the third digit is 5 or above^12^.

### Results on simulated data

Table 6 lists the image reconstruction metric results of the FCD on the 40,000 simulated data points. For the metrics MS-SSIM, PSNR, and the Dice coefficient, higher values indicate better performance; for LPIPS and Hausdorff distance, lower values are preferable. All metrics shown in the table demonstrate favorable and promising results for the FCD, with some metrics, such as MS-SSIM, approaching the upper margin of performance. Most importantly, these results indicate that the FCD was well-aligned with the dataset, proving effective in learning and accurately reconstructing the data.

**Table 6:** Image reconstruction experiment results on 40,000 simulated data samples

Metrics	FCD
MS-SSIM	0.97 ± 0.02
LPIPS	0.04 ± 0.03
PSNR	35.60 ± 4.06
Dice Coefficient	0.84 ± 0.08
Hausdorff Distance	4.80 ± 5.61

The results presented after this point in this section are based on a subset of simulated data comprising 1000 samples, which includes the STIX image reconstruction algorithms set alongside FCD. This subset consists of 250 samples for each source count, ranging from 1 to 4, totaling 1000 samples. It was constructed by selecting the first 250 data samples from each corresponding source group in the larger simulated dataset.

Table 7 presents the image reconstruction results of all the imaging algorithms discussed in this research on the 1000 simulated data samples. The FCD achieves the best overall performance, with VIS_FWDFIT producing very close results, trailing slightly behind based on the standard deviation in the MS-SSIM and Dice coefficient metrics. The reason VIS_FWDFIT achieved such high results is due to its parametric nature, which allows it to perfectly match the count and locations of the sources when the expected number of sources is provided. In these results, VIS_FWDFIT was run with the expected number of sources.

**Table 7:** Image reconstruction experiment results on 1000 simulated data samples compared with other image reconstruction algorithms

Metrics	FCD	VIS_FWDFIT	MEM_GE	CLEAN
MS-SSIM	**0**.**97** ± **0**.**02**	0.97 ± 0.03	0.89 ± 0.07	0.95 ± 0.03
LPIPS	**0**.**04** ± **0**.**03**	0.05 ± 0.04	0.07 ± 0.04	0.11 ± 0.04
PSNR	**35**.**70** ± **3**.**97**	35.50 ± 4.78	32.22 ± 3.93	31.77 ± 3.26
Dice Coefficient	**0**.**83** ± **0**.**08**	0.83 ± 0.10	0.76 ± 0.10	0.76 ± 0.11
Hausdorff Distance	**5**.**08** ± **6**.**26**	5.40 ± 7.33	34.46 ± 33.44	7.16 ± 6.56

**Fig. 8:**
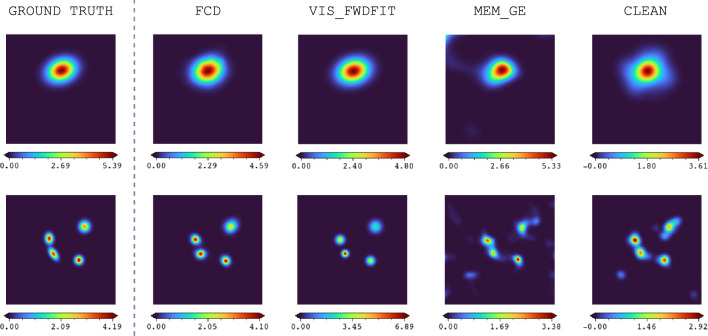
Ground truth vs. image reconstruction results of all imaging algorithms discussed in this work.

Based on the results in Table 7, where the FCD achieves the highest PSNR value, it can also be inferred that the FCD introduces the least amount of noise compared to the other image reconstruction algorithms. Two examples, featuring one and four sources, comparing the ground truth in the simulated data with the results from all imaging algorithms, are shown in Fig. 8.

### Results on observational data

Table 8 presents the results of the data reconstruction experiments conducted on observational data, comparing the visibilities obtained from the STIX instrument with those predicted from the reconstructed images produced by the imaging algorithms used in this research. For the metrics MAE, spectral convergence, and ${\chi }^2$, lower values indicate better performance, while for cosine similarity, higher values are preferable. In this context, “better” indicates greater similarity between the predicted and observed visibility vectors.

**Table 8:** Data reconstruction experiment results on observational data

Metrics	FCD	VIS_FWDFIT	MEM_GE	CLEAN
MAE	0.55 ± 2.78	0.59 ± 2.95	**0**.**42** ± **2**.**11**	0.46 ± 2.04
Cosine Similarity	0.94 ± 0.05	0.94 ± 0.06	**0**.**97** ± **0**.**03**	0.95 ± 0.05
Spectral Convergence	0.31 ± 0.13	0.32 ± 0.14	**0**.**27** ± **0**.**10**	0.32 ± 0.16
${\chi }^2$	3.54 ± 5.70	3.63 ± 4.22	**2**.**14** ± **1**.**64**	2.98 ± 3.01

**Fig. 9:**
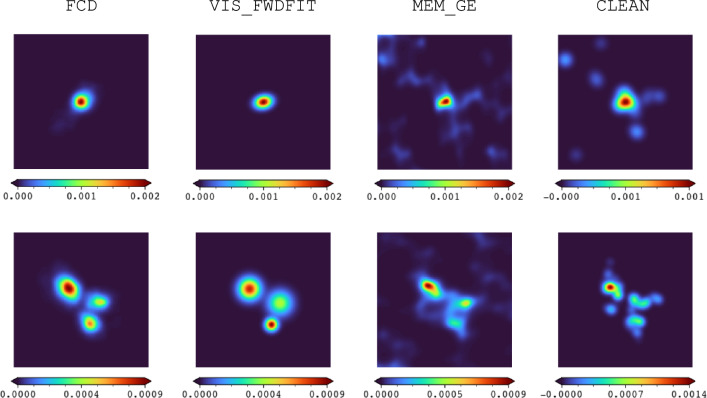
Comparison of image reconstruction algorithms and FCD involving one and three sources on observational data.

MEM_GE achieves the leading results among all the methods compared in data reconstruction, as shown in Table 8. A visual comparison of the reconstructed image quality between the FCD and the STIX imaging methods is presented in Fig. 9, featuring examples with one and three sources.

The chi-squared (${\chi }^2$) statistic is a measure that accounts for the experimental uncertainty in the observational data. For the ${\chi }^2$ metric, lower values are preferable, with values closer to 0 indicating a better fit. Ideally, a “good” reconstruction should have a ${\chi }^2$ value close to 1, implying that the reconstruction is consistent with the data without overfitting the noise. However, Table 8 shows mean ${\chi }^2$ values that are consistently higher than 1 for all methods. This is likely due to the fact that, in addition to statistical errors, observational STIX data are affected by systematic errors. The current version of the STIX data analysis pipeline adds $5\%$ systematic errors in quadrature to the statistical ones [17]. However, this value is rather arbitrary and could lead to underestimated $\sigma$ values in Eq. 11. Because of the intrinsic uncertainty on systematic errors, it is difficult to interpret ${\chi }^2$ values in a strict probabilistic sense [53]. Therefore, the ${\chi }^2$ values provided in Table 8 should be considered primarily for comparison between the different methods.

**Fig. 10:**
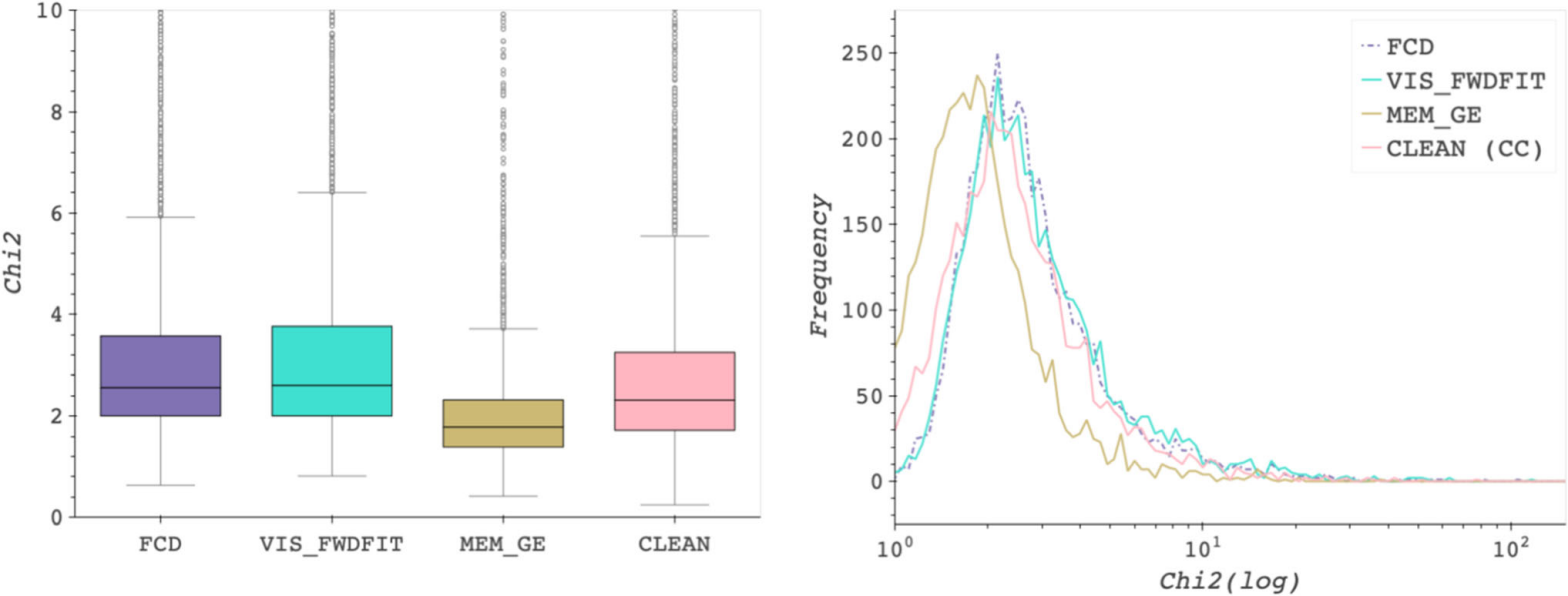
Comparison of chi-squared values (*Chi2* in the graphics) across imaging algorithms: zoomed-in view up to a value of 10 highlighting outliers as hollow gray points *(left)* and frequency-score plot (log scale) showing overall results *(right)*.

The distribution and spread of the ${\chi }^2$ results in Table 8 can also be observed in Fig. 10, where the box-and-whisker plot on the right offers a zoomed-in view, while the line plot on the left provides an overall view of the data distribution on a logarithmic scale.

Box-and-whisker plots provide a visual summary of data distribution by displaying the median, quartiles, and potential outliers. The *box* represents the interquartile range (IQR), containing the middle 50% of the data, while the *whiskers* extend to the smallest and largest values within 1.5 times the IQR from the lower and upper quartiles, respectively. Any data points beyond the whiskers are considered outliers and are marked individually, providing insights into the data’s spread and variability. The median values of FCD, VIS_FWDFIT, MEM_GE, and CLEAN are 2.56, 2.61, 1.78, and 2.31 respectively, as shown on the right side of the Fig. 10. The outliers are indicated by hollow gray points in the figure.

The line plot in Fig. 10 shows the distribution of the ${\chi }^2$ values on a logarithmic scale. The horizontal axis represents the ${\chi }^2$ values, while the vertical axis indicates the frequency of occurrences within the dataset. Both plots in the figure demonstrate that MEM_GE achieves the best results, followed by CLEAN in second place. FCD performs slightly better than VIS_FWDFIT, placing third.

### Evaluation of runtime

It should be noted that while our deep learning-based solution, FCD, runs on Python, the remaining image reconstruction algorithms are executed in IDL^13^. Python tends to have higher memory usage and longer runtimes compared to compiled languages like IDL, especially for large-scale numerical operations. On the other hand, IDL, a language optimized for scientific data analysis, is typically faster in execution for certain specialized tasks due to its built-in array processing capabilities, though it can be less flexible and more memory-intensive for larger datasets [54].

In general, Python’s performance can be enhanced by leveraging libraries such as NumPy, Numba, and TensorFlow, which are optimized for scientific computing, but IDL’s native strengths in handling array-based data and faster execution time make it a more common choice in astronomy and other scientific domains for image reconstruction tasks. Python’s wide ecosystem and flexibility in handling modern machine learning workflows make it the preferred choice for our neural network model. We support this choice by using the aforementioned libraries, giving our solution the best chance of competing with IDL-based algorithms in terms of runtime. Additionally, since the imaging algorithms run on a Central Processing Unit (CPU) using IDL software, the FCD was also run on the same Apple M1 CPU within a single-threaded environment to ensure a fair comparison of the results in Table 9.

**Table 9:** Time-to-solution comparison in seconds between the FCD and the STIX image reconstruction algorithms for a single reconstructed image

Imaging Algorithm	Time-to-Solution (s)
VIS_FWDFIT	5.522 ± 3.655
MEM_GE	9.048 ± 6.844
**CLEAN**	**0**.**032** ± **0**.**092**
**FCD**	**0**.**032** ± **0**.**005**

Time-to-solution values for the STIX image reconstruction algorithms and the FCD are listed in Table 9. These values were obtained by reconstructing images on the 1000-sample dataset and calculating the mean runtime for each algorithm,^14^ ultimately providing the mean runtime for a single-image reconstruction. The table shows that both CLEAN and FCD offer solutions in milliseconds (ms), with FCD outperforming CLEAN by exhibiting a slightly lower standard deviation.

The runtime of these algorithms becomes particularly crucial given that the STIX dataset has accumulated more than 50,000 data points, and this number continues to grow. Without acceleration mechanisms to handle the high influx of data, runtime increases proportionally with data volume. To address this, we conducted an additional experiment on the FCD, accelerated using a single graphics processing unit (GPU), to observe its performance when processing data samples ranging from 1 to 10,000 at once. Table 10 presents the runtime results for these different scenarios. The results demonstrate that processing larger datasets leads to efficient reconstruction times, with 10,000 visibilities being reconstructed in approximately 2 s. Additionally, it can be noted that the runtime for reconstructing one image is very close to that for reconstructing ten images. This is because, in these cases, the data transfer time between the CPU and GPU dominates the execution time, resulting in very similar runtimes. This also explains the faster result of the FCD in Table 9 compared to the single-image reconstruction result in Table 10 when using the GPU versus the CPU. It is important to mention that all runtime experiments of the FCD were conducted within the complete framework of our proposed system, as illustrated in Fig. 6 and previously discussed in Sect. 2.3.2.

**Table 10:** Time-to-solution of the FCD in different runtime scenarios

FCD	Time-to-Solution (s)
Runtime-1	0.035 ± 0.006
Runtime-10	0.036 ± 0.010
Runtime-100	0.051 ± 0.008
Runtime-1000	0.240 ± 0.010
Runtime-10000	2.069 ± 0.039

Finally, we note that existing imaging methods for reconstructing STIX visibilities, i.e., VIS_FWDFIT, MEM_GE, and CLEAN, are not designed for simultaneous multiple-image reconstructions, unlike the proposed FCD. Running these algorithms in parallel would require separate CPU cores, which is not yet implemented. Additionally, their IDL-based implementations are not optimized for GPU acceleration. In contrast, FCD can process multiple visibility datasets simultaneously and leverage GPUs when available, making it the most suitable approach for large-scale STIX data processing. For instance, reconstructing a single image for each of the approximately 50,000 flaring events observed by STIX so far would take at most around 10 s on a GPU (see Table 10). In comparison, based on results in Table 9, VIS_FWDFIT, MEM_GE, and CLEAN would require approximately 76 h, 125 h, and 26 min, respectively, for the same task^15^.

### Discussion

We have achieved satisfactory results, providing a fast, parameter-free solution that minimizes artifacts in image reconstruction, as demonstrated in the previous sections, on both simulated and observational data. We trained our proposed FCD on synthetic data generated by a simulation code that mimics the behavior of an *ideal* STIX instrument, meaning that any (unknown) systematic instrumental errors are not considered in the simulation process. The simulator relies on analytical computations of the number of counts recorded by STIX corresponding to X-ray Gaussian sources, for which a closed-form solution is known. This approach makes the simulation computationally efficient. For instance, generating a random dataset containing 1000 data points takes approximately 1.4 s^16^. As shown in Sect. 6.2, the FCD, despite being solely trained on simulated data, demonstrated robustness in handling differences between simulated and observational data. While it ranked third among four methods on the observational dataset, it still provided reliable results in the majority of cases.

**Fig. 11:**
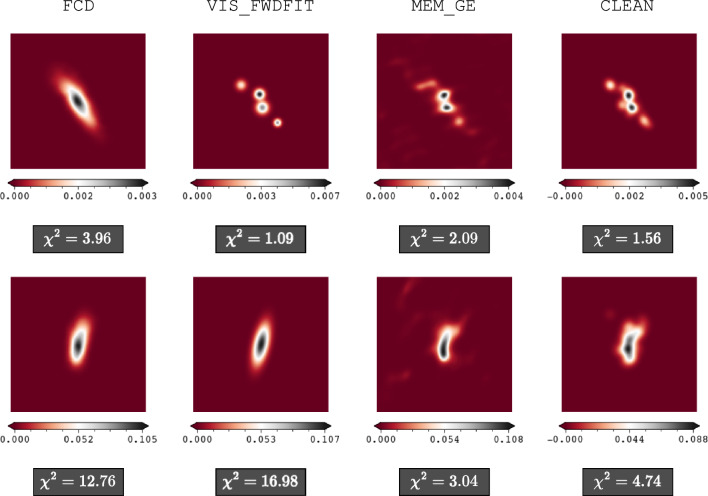
Examples illustrating the limitations of the FCD in handling closely spaced sources *(top)* and elongated/asymmetrical sources *(bottom)* in observational data. Additionally, the chi-squared (${\chi }^2$) metric results for each image are provided underneath.

We found that the FCD underperformed on observational data when sources were either too close to one another or elongated but asymmetrical, as shown in Fig. 11. For both images reconstructed by the FCD in the figure, we observe that the orientation, flux intensity range, and overall dimensions of the X-ray emission are consistent with those retrieved by the other algorithms. The FCD also introduces visibly less artifacts into the image compared to MEM_GE and CLEAN, especially in the bottom example. However, in the top example of Fig. 11, the FCD fails to distinguish between closely spaced sources, reconstructing a single, larger source. In the bottom example, the FCD reconstructs a shape similar to the elongated Gaussian retrieved by VIS_FWDFIT and fails to reproduce the non-symmetric source captured by MEM_GE and CLEAN. The sub-optimal performance of the FCD results in generally higher $\chi ^2$ values compared to the other methods, with the only exception being the VIS_FWDFIT reconstruction in the bottom row. This indicates that the network, as anticipated, struggles to accurately reconstruct shapes that were either not present or minimally represented in the training set.

The decision to train the FCD exclusively on simulated data was made to allow it to develop its own unique identity, much like the other STIX image reconstruction algorithms, and to produce images with a minimum amount of artifacts. A possible strategy to improve the generalization capabilities of our proposed FCD on observational data is to make the current version of the STIX data simulator more realistic. For instance, (unknown) instrumental errors affecting the data can be simulated by randomly perturbing the visibility amplitude and phase values, independent of the magnitude of statistical errors. These systematic errors would be in addition to statistical errors, which are already accounted for in the simulation procedure adopted for this study. Further, more realistic source shapes, beyond the currently adopted Gaussian functions, could be explored. However, defining a more appropriate parametrization of a flaring source shape remains a challenging task that has not yet been addressed in the context of STIX imaging.

In an alternative approach, if the goal is not to achieve unique image reconstructions, the images provided by one of the existing STIX imaging algorithms could be used as training data. However, as the images reconstructed from real STIX data are typically affected by spurious artifacts introduced by the adopted image reconstruction techniques, it would be highly impractical to obtain large datasets of observational STIX data and corresponding high-quality images. Indeed, an expert user would need to carefully check the reliability of each reconstructed image. To overcome this limitation, a small dataset of selected high-quality images reconstructed from observational STIX data could be created and utilized for transfer learning strategies. We expect that pre-training the model on simulated STIX data and fine-tuning it using a small dataset of observational data would improve model performance. This approach, however, requires considerable effort to construct an appropriate dataset, which is beyond the scope of our current exploratory study.

## Conclusion

This work introduces a deep learning-based method for reconstructing solar flares, distinguishing itself through its efficiency by leveraging a compact model and operating in the frequency domain. The proposed neural architecture, presented in Sect. 3, is an overcomplete supervised autoencoder designed to take a compressed form of data-specifically, Fourier components (visibilities)-and reconstruct higher-dimensional images of solar flares. Inspired by the operational mechanism of the architecture, we have named the autoencoder the Fourier convolutional decoder (FCD).

The primary objective of this work is to efficiently reconstruct images with minimal delays and artifacts, in a parameter-free, directly runnable, i.e., end-to-end, manner. To achieve this, the FCD was trained exclusively on simulated data. We utilized both simulated and observational data from the STIX instrument on the solar orbiter in this work, with all data-related aspects analyzed in Sect. 2.3.

Evaluation was conducted using two different sets of metrics defined in Sect. 5: image reconstruction metrics, which compare the ground truth images with the reconstructed images, and data reconstruction metrics, which compare the input visibilities to the visibilities predicted from the reconstructed images. The first set of metrics was applied to simulated data, while the second set was used on observational data. In both cases, FCD was compared against three established STIX imaging algorithms, selected for their representative value. These imaging algorithms were introduced and compared in Sect. 2.2. In addition to reconstruction-based experiments, the runtime of the algorithms was measured and compared. The results presented in Sect. 6 demonstrate that the FCD achieved favorable outcomes overall, despite being trained exclusively on simulated data. The FCD outperforms the other algorithms in image reconstruction, as shown in Table 7, and further demonstrated the least noise in the reconstructed images, as indicated by the PSNR metric. In the runtime experiments explained in Sect. 6.3, while CLEAN and FCD achieve comparable efficiency when reconstructing a single image (Table 9), the benefits of the underlying mechanisms of the FCD become apparent when reconstructing multiple images, scaling up to thousands, as shown in Table 10.

One of the recurrent challenges in the application of machine learning is the transition from simulated to observational data. While simulated data are useful for training and validation, they often lack the variability and complexity of real-world data, resulting in models that perform well in controlled environments but struggle in real-world scenarios.

In transitioning from the “simulated world” to the “real-world,” issues such as noise, unexpected artifacts, and incomplete data can lead to diminished model performance. Our approach addresses these challenges by designing a neural network that, while trained exclusively on simulated data, was developed with both data types in mind. As reflected in the results in Sect. 6, the FCD performed competitively, ranking among the top algorithms, but also showed areas for improvement in handling real-world variability.

Section 6.4 elaborates on edge cases where the FCD underperformed in real-world scenarios using observational data. We discuss the possible causes of these cases in that section and suggest that they can be addressed by adjusting the simulator and/or applying transfer learning with fine-tuning on a carefully curated small set of observational data.

Even though our initial application focuses on solar physics, where reconstructing solar flare images from X-ray data presents unique challenges, the methodology we introduce has broader applicability. For instance, in medical imaging, particularly in magnetic resonance imaging (MRI), Fourier transforms play a key role in converting frequency domain data, i.e., *k*-space, into spatial domain images [55]. While the data and reconstruction techniques differ, the underlying principle of using Fourier components to enhance image reconstruction remains relevant [56, 57].

In conclusion, we demonstrated the effectiveness of the FCD deep learning model in solar flare image reconstruction from Fourier components, highlighting its accuracy, efficiency, and deployability within this work. The adaptability of our autoencoder approach suggests potential benefits not only in this domain, but also in other fields, provided the method is tailored to address the specific challenges of the data and reconstruction processes in those areas. Future work will focus on developing a more robust model for real-world applications, and validating the model’s ability to adapt to different domain-specific data.

## Data Availability

The simulated data used in this research can be reproduced using the code available at https://github.com/i4Ds/stix-data-generator. The observational data are publicly accessible through the STIX Data Center at https://datacenter.stix.i4ds.net.

## Appendix A Software and library details

We use Python^17^ as the programming language and TensorFlow [58] as a deep learning library. The other main software libraries we use are as follows. The full list of software requirements along with the versions can be found in our GitHub repositories.

- SciPy [59] and Astropy[60, 61]: Processing of STIX observational data.
- NumPy [62]: Implementation of the STIX simulator; metrics Dice Coefficient, Spectral Convergence, chi-squared (${\chi }^2$); and application of any numerical calculations including arrays.
- scikit-image [63]: Application of MS-SSIM, PSNR, and Hausdorff Distance.
- scikit-learn [64]: Application of Cosine Similarity and MAE metrics.
- OpenCV^18^: Image-related operations such as resizing and color-scheme changing such as binarization.
- matplotlib [65], bokeh,^19^HoloViews^20^: Plotting and visualizations.

## Appendix B Data access and reference links

The observational data used in this paper are publicly available and can be accessed through the STIX Data Center.^21^ Table 11 provides access links to the datasets corresponding to the figures presented in this paper, ensuring reproducibility and facilitating further analysis. Each figure reference is accompanied by a direct link to the data used for that specific figure.

**Table 11:** Links to observational data used in this paper, with corresponding UIDs. Each link corresponds to the dataset referenced in the specified figure

Figure Reference	Data Access Link	UID
Fig. 2	https://datacenter.stix.i4ds.net/view/image-archive/report/4300.	2110090089
Fig. 3—top row	https://datacenter.stix.i4ds.net/view/list/bsd/id/13428.	2207262350
Fig. 3—second to top row	https://datacenter.stix.i4ds.net/view/list/bsd/id/4986.	2110280004
Fig. 3—third to top row	https://datacenter.stix.i4ds.net/view/list/bsd/id/14129.	2208284257
Fig. 3—bottom row	https://datacenter.stix.i4ds.net/view/image-archive/report/2108.	2109230031
Fig. 5	https://datacenter.stix.i4ds.net/view/image-archive/report/30900.	2303295417
Fig. 9—single source	https://datacenter.stix.i4ds.net/view/image-archive/report/28839.	2301232231
Fig. 9—three sources	https://datacenter.stix.i4ds.net/view/list/bsd/id/14129.	2208284257
Fig. 11—top example	https://datacenter.stix.i4ds.net/view/image-archive/report/2108.	2109230031
Fig. 11—bottom example	https://datacenter.stix.i4ds.net/view/list/bsd/id/21603.	2305116227

Alternatively, the images can also be accessed using the Unique Identifier (UID) information via the STIX Data Center. To retrieve the data, follow the path:

STIX Data Center $\rightarrow$ Science Data Browser^22^. $\rightarrow$ Search using the UID.

## Footnotes

1 The complete model is stored in a .keras file, while the weights are stored separately in a .h5 file. Their sizes are measured in memory, excluding any file structure overhead.

2 The inherent limitations of the instruments, the incomplete and noisy nature of the data, and the complexity of solar phenomena all contribute to the challenge of obtaining artifact- and noise-free images in solar physics. Therefore, our aim in this study is to reconstruct the images with minimal artifacts, as effectively as possible.

3 This dataset is detailed in Sect. 6. The “Time-to-Solution” data in Table 1 will be updated to include our image reconstruction approach, with details of the experiment and a discussion of the results in Sect. 6.3.

4 Due to the linearity of the Fourier transform, the visibilities of a sum of functions are equivalent to the sum of the visibilities of each individual component.

5 This number reflects the final data download as of 29/05/2024.

6 The 20% noise level is calculated based on the expected Poisson distribution of the photon counts, where the standard deviation is equal to the square root of the number of counts. For counts greater than 5000, the relative noise level is lower than 20%.

7 The thresholds of 5000 photon counts and 5 min integration time were empirically determined based on expert consultation.

8 The layered view of the FCD architecture was visualized using the open-source tool VisualKeras [38].

9 This range is [$-$34513.254, 38953.734] before normalization and [$-$2.0, 2.0] after normalization in the training set.

10 The visibilities illustrated here are observed data and are the same as those in Fig. 2.

11 The statistical uncertainty on the visibility amplitude is obtained by propagating the statistical errors on the measured detector counts (either simulated or observational), which are corrupted by Poisson noise (see Sect. 2.3.1).

12 Except for the runtime experiments in Sect. 6.3 where rounding occurs after three decimal places due to the small numbers involved.

13 Among the compared algorithms, only the CLEAN and MEM_GE algorithms have a Python version implemented for X-ray data, available at https://github.com/samaloney/xrayvision. Given the common practice of using IDL-based algorithms in this field, we opted to compare with the IDL versions of these methods.

14 The initial runtime of FCD in the runtime experiments was disregarded because it includes the time required for the just-in-time compilation of certain functions, which are optimized during the first run and do not require recompilation in subsequent runs.

15 An estimate of the computational time for VIS_FWDFIT, MEM_GE, and CLEAN is obtained by multiplying the mean values in Table 9 by 50,000. However, the estimated runtime for VIS_FWDFIT decreases if image reconstruction is limited to configurations with 1 or 2 sources, rather than including configurations with up to 4 sources as presented in Table 9.

16 The mean time, 1.399, is measured as the average of 10 runs, with a standard deviation of 0.102. The code to generate the dataset was executed in a single-threaded environment on an Apple M2 Pro CPU.

## Code availability

The code used to build, train, and evaluate the deep learning model, as well as the metrics used in this research, is available at https://github.com/i4Ds/fcd.

## Declarations

### Model Availability

The autoencoder model developed for this research is available for download at https://huggingface.co/mervess/FCD-Solar. Additionally, a demonstration of the model can be accessed at https://huggingface.co/spaces/mervess/FCD-Solar-Demo.

## References

[1] Yaqub M, Jinchao F, Arshid K, Ahmed S, Zhang W, Nawaz MZ, Mahmood T (2022) Deep learning-based image reconstruction for different medical imaging modalities. Comput Math Methods Med 2022(1):8750648. 10.1155/2022/8750648 35756423 10.1155/2022/8750648 PMC9225884

[2] Wu D, Li S, Yang J, Sawan M (2024) Neuro-BERT: rethinking masked autoencoding for self-supervised neurological pretraining. IEEE J Biomed Health Inf. 10.1109/JBHI.2024.3415959 10.1109/JBHI.2024.3415959 38889028

[3] Taran O, Bait O, Dessauges-Zavadsky M, Holotyak T, Schaerer D, Voloshynovskiy S (2023) Challenging interferometric imaging: machine learning-based source localization from UV-plane observations. Astron Astrophys 674:161. 10.1051/0004-6361/202245778

[4] Chen S, Guo W (2023) Auto-encoders in deep learning-a review with new perspectives. Mathematics 11(8):1777. 10.3390/math11081777

[5] Díaz Baso CJ, De La Cruz Rodríguez J, Danilovic S (2019) Solar image denoising with convolutional neural networks. Astron Astrophys 629:99. 10.1051/0004-6361/201936069

[6] Xu L, Sun W-Q, Yan Y-H, Zhang W-Q (2020) Solar image deconvolution by generative adversarial network. Res Astron Astrophys 20(11):170. 10.1088/1674-4527/20/11/170

[7] Xu L, Yan Y, Huang X (2022) Deep learning in solar image generation tasks. Deep learning in solar astronomy. Springer, Singapore, pp 59–81

[8] Geyer F, Schmidt K, Kummer J, Brüggen M, Edler HW, Elsässer D, Griese F, Poggenpohl A, Rustige L, Rhode W (2023) Deep-learning-based radiointerferometric imaging with GAN-aided training. Astron Astrophys 677:167. 10.1051/0004-6361/202347073

[9] Aghabiglou A, Chu CS, Dabbech A, Wiaux Y (2024) The R2D2 deep neural network series paradigm for fast precision imaging in radio astronomy. Astrophys J Suppl Ser 273(1):3. 10.3847/1538-4365/ad46f5

[10] Xia Y, Su Y, Liu H, Yu W, Li Z, Chen W, Huang Y, Gan W (2024) A new solar hard X-ray image reconstruction algorithm for ASO-S/HXI based on deep learning. Sol Phys 299(11):158. 10.1007/s11207-024-02399-4

[11] Felix S, Bolzern R, Battaglia M, Csillaghy A (2019) Solar image reconstruction from visibilities with compressed sensing. Astronom Data Anal Softw Syst XXVI 521:358

[12] Duval-Poo MA, Piana M, Massone AM (2018) Solar hard X-ray imaging by means of compressed sensing and finite isotropic wavelet transform. Astron Astrophys 615:59. 10.1051/0004-6361/201731765

[13] Massa P, Schwartz R, Tolbert AK, Massone AM, Dennis BR, Piana M, Benvenuto F (2020) MEM_ge: a new maximum entropy method for image reconstruction from solar X-Ray visibilities. Astrophys J 894(1):46. 10.3847/1538-4357/ab8637

[14] Benz AO (2016) Flare observations. Living Rev Sol Phys 14(1):2. 10.1007/s41116-016-0004-3 10.12942/lrsp-2008-1 PMC4841183 27194959

[15] Müller D, Cyr OCS, Zouganelis I, Gilbert HR, Marsden R, Nieves-Chinchilla T, Antonucci E, Auchère F, Berghmans D, Horbury TS, Howard RA, Krucker S, Maksimovic M, Owen CJ, Rochus P, Rodriguez-Pacheco J, Romoli M, Solanki SK, Bruno R, Carlsson M, Fludra A, Harra L, Hassler DM, Livi S, Louarn P, Peter H, Schühle U, Teriaca L, Iniesta JCdT, Wimmer-Schweingruber RF, Marsch E, Velli M, Groof AD, Walsh A, Williams D (2020) The solar orbiter mission: science overview. Astron Astrophys. 10.1051/0004-6361/202038467

[16] ...Krucker S, Hurford GJ, Grimm O, Kögl S, Gröbelbauer H-P, Etesi L, Casadei D, Csillaghy A, Benz AO, Arnold NG, Molendini F, Orleanski P, Schori D, Xiao H, Kuhar M, Hochmuth N, Felix S, Schramka F, Marcin S, Kobler S, Iseli L, Dreier M, Wiehl HJ, Kleint L, Battaglia M, Lastufka E, Sathiapal H, Lapadula K, Bednarzik M, Birrer G, Stutz S, Wild C, Marone F, Skup KR, Cichocki A, Ber K, Rutkowski K, Bujwan W, Juchnikowski G, Winkler M, Darmetko M, Michalska M, Seweryn K, Białek A, Osica P, Sylwester J, Kowalinski M, Ścisłowski D, Siarkowski M, Steslicki M, Mrozek T, Podgórski P, Meuris A, Limousin O, Gevin O, Mer IL, Brun S, Strugarek A, Vilmer N, Musset S, Maksimović M, Fárník F, Kozáček Z, Kašparová J, Mann G, Önel H, Warmuth A, Rendtel J, Anderson J, Bauer S, Dionies F, Paschke J, Plüschke D, Woche M, Schuller F, Veronig AM, Dickson ECM, Gallagher PT, Maloney SA, Bloomfield DS, Piana M, Massone AM, Benvenuto F, Massa P, Schwartz RA, Dennis BR, Beek HFV, Rodríguez-Pacheco J, Lin RP (2020) The spectrometer/telescope for imaging X-rays (STIX). Astron Astrophys 642:15. 10.1051/0004-6361/201937362

[17] Xiao H, Maloney S, Krucker S, Dickson E, Massa P, Lastufka E, Francesco Battaglia A, Etesi L, Hochmuth N, Schuller F, Ryan DF, Limousin O, Collier H, Warmuth A, Piana M (2023) The data center for the spectrometer and telescope for imaging X-rays (STIX) on board solar orbiter. Astron Astrophys 673:142. 10.1051/0004-6361/202346031

[18] Lemen JR, Title AM, Akin DJ, Boerner PF, Chou C, Drake JF, Duncan DW, Edwards CG, Friedlaender FM, Heyman GF, Hurlburt NE, Katz NL, Kushner GD, Levay M, Lindgren RW, Mathur DP, McFeaters EL, Mitchell S, Rehse RA, Schrijver CJ, Springer LA, Stern RA, Tarbell TD, Wuelser J-P, Wolfson CJ, Yanari C, Bookbinder JA, Cheimets PN, Caldwell D, Deluca EE, Gates R, Golub L, Park S, Podgorski WA, Bush RI, Scherrer PH, Gummin MA, Smith P, Auker G, Jerram P, Pool P, Soufli R, Windt DL, Beardsley S, Clapp M, Lang J, Waltham N (2012) The atmospheric imaging assembly (AIA) on the solar dynamics observatory (SDO). Sol Phys 275(1):17–40. 10.1007/s11207-011-9776-8

[19] Tandberg-Hanssen E, Emslie AG (1988) The physics of solar flares, vol 14. Cambridge University Press, Cambridge

[20] Brown JC (1971) The deduction of energy spectra of non-thermal electrons in flares from the observed dynamic spectra of hard X-Ray bursts. Sol Phys 18:489–502. 10.1007/BF00149070

[21] Krucker S, Christe S, Glesener L, Ishikawa S-n, Ramsey B, Takahashi T, Watanabe S, Saito S, Gubarev M, Kilaru K, Tajima H, Tanaka T, Turin P, McBride S, Glaser D, Fermin J, White S, Lin R (2014) First images from the focusing optics X-ray solar imager. Astrophys J 793:32. 10.1088/2041-8205/793/2/L32

[22] Prince TA, Hurford GJ, Hudson HS, Crannell CJ (1988) Gamma-ray and hard X-ray imaging of solar flares. Sol Phys 118(1):269–290. 10.1007/BF00148596

[23] Hurford GJ (2013) X-ray imaging with collimators, masks and grids. Observing photons in space: a guide to experimental space astronomy. Springer, New York, pp 243–254

[24] Massa P, Hurford GJ, Volpara A, Kuhar M, Battaglia AF, Xiao H, Casadei D, Perracchione E, Garbarino S, Guastavino S, Collier H, Dickson ECM, Emslie AG, Ryan DF, Maloney SA, Schuller F, Warmuth A, Massone AM, Benvenuto F, Piana M, Krucker S (2023) The STIX imaging concept. Sol Phys 298(10):114. 10.1007/s11207-023-02205-7

[25] Piana M, Emslie AG, Massone AM, Dennis BR (2022) Hard X-ray imaging of solar flares. Springer, Cham

[26] Müller H, Massa P, Mus A, Kim J-S, Perracchione E (2024) Identifying synergies between VLBI and STIX imaging. Astron Astrophys 684:47. 10.1051/0004-6361/202348040

[27] Dickson E, Richard, Etesi LI, paolomassa, Schuller F, Maloney S, Battaglia A, Hochmuth N, annavolp, Benvenuto F, testinguser (2023) tomekmrozek: i4Ds/STIX-GSW: Release of v0.5.1 - Bugfix Release. Zenodo. https://zenodo.org/records/10078466 Accessed 2024-08-20

[28] Volpara A, Massa P, Perracchione E, Battaglia AF, Garbarino S, Benvenuto F, Krucker S, Piana M, Massone AM (2022) Forward fitting STIX visibilities. Astron Astrophys 668:145. 10.1051/0004-6361/202243907

[29] Kennedy J, Eberhart R (1995) Particle swarm optimization. In: Proceedings of ICNN’95 - international conference on neural networks 4:1942–19484. 10.1109/ICNN.1995.488968

[30] Högbom JA (1974) Aperture synthesis with a non-regular distribution of interferometer baselines. Astron Astrophys, Suppl Ser 15:417

[31] Massa P, Perracchione E, Garbarino S, Battaglia AF, Benvenuto F, Piana M, Hurford G, Krucker S (2021) Imaging from STIX visibility amplitudes. Astron Astrophys 656:25. 10.1051/0004-6361/202140946

[32] Stiefel MZ, Battaglia AF, Barczynski K, Collier H, Volpara A, Massa P, Schwanitz C, Tynelius S, Harra L, Krucker S (2023) Solar flare hard X-rays from the anchor points of an eruptive filament. Astron Astrophys 670:89. 10.1051/0004-6361/202245044

[33] Li P, Pei Y, Li J (2023) A comprehensive survey on design and application of autoencoder in deep learning. Appl Soft Comput 138:110176. 10.1016/j.asoc.2023.110176

[34] Zeiler MD, Krishnan D, Taylor GW, Fergus R (2010) Deconvolutional networks. In: 2010 IEEE computer society conference on computer vision and pattern recognition, pp. 2528–2535. IEEE, San Francisco, CA, USA. 10.1109/CVPR.2010.5539957

[35] Dumoulin V, Visin F (2016) A guide to convolution arithmetic for deep learning. arXiv. 10.48550/arXiv.1603.07285

[36] Gao H, Yuan H, Wang Z, Ji S (2017) Pixel deconvolutional networks. arXiv. 10.48550/arXiv.1705.06820

[37] Jacot A, Gabriel F, Ged F, Hongler C (2022) Freeze and chaos: NTK views on DNN normalization, checkerboard and boundary artifacts. In: Mathematical and Scientific Machine Learning, pp. 257–270

[38] Gavrikov P (2024) paulgavrikov/visualkeras. https://github.com/paulgavrikov/visualkeras Accessed 2024-07-15

[39] He K, Zhang X, Ren S, Sun J (2015) Delving deep into rectifiers: surpassing human-level performance on imagenet classification. In: 2015 IEEE International conference on computer vision (ICCV), pp. 1026–1034. IEEE, Santiago, Chile. 10.1109/ICCV.2015.123

[40] Hendrycks D, Gimpel K (2023) Gaussian error linear units (GELUs). arXiv. 10.48550/arXiv.1606.08415

[41] Ba JL, Kiros JR, Hinton GE (2016) Layer normalization. arXiv. 10.48550/arXiv.1607.06450

[42] Ioffe S, Szegedy C (2015) Batch normalization: accelerating deep network training by reducing internal covariate shift. In: Proceedings of the 32nd international conference on machine learning, pp. 448–456

[43] Kumar A, Sodhi SS (2020) Comparative analysis of gaussian filter, median filter and denoise autoenocoder. In: 2020 7th international conference on computing for sustainable global development (INDIACom), pp. 45–51. 10.23919/INDIACom49435.2020.9083712

[44] So CW, Yuen ELH, Leung EHF, Pun JCS (2024) Solar image quality assessment: a proof of concept using variance of Laplacian method and its application to optical atmospheric condition monitoring. Publ Astron Soc Pac 136(4):044504. 10.1088/1538-3873/ad3b39

[45] Wang X (2007) Laplacian operator-based edge detectors. IEEE Trans Pattern Anal Mach Intell 29(5):886–890. 10.1109/TPAMI.2007.1027 17356206 10.1109/TPAMI.2007.1027

[46] Kingma DP, Ba J (2017) Adam: a method for stochastic optimization. arXiv. 10.48550/arXiv.1412.6980

[47] Liao P-S, Chen T-S, Chung P-C (2001) A fast algorithm for multilevel thresholding. J Inf Sci Eng 17(5):713–727. 10.6688/JISE.2001.17.5.1

[48] Wang Z, Bovik AC, Sheikh HR, Simoncelli EP (2004) Image quality assessment: from error visibility to structural similarity. IEEE Trans Image Process 13(4):600–612. 10.1109/TIP.2003.819861 15376593 10.1109/tip.2003.819861

[49] Wang Z, Simoncelli EP, Bovik AC (2003) Multiscale structural similarity for image quality assessment. In: The Thrity-Seventh asilomar conference on signals, systems & computers, 2003, pp. 1398–1402. IEEE, Pacific Grove, CA, USA. 10.1109/ACSSC.2003.1292216

[50] Zhang R, Isola P, Efros AA, Shechtman E, Wang O (2018) The unreasonable effectiveness of deep features as a perceptual metric. In: 2018 IEEE/CVF Conference on computer vision and pattern recognition, pp. 586–595. IEEE, Salt Lake City, UT. 10.1109/CVPR.2018.00068

[51] Huttenlocher DP, Klanderman GA, Rucklidge WJ (1993) Comparing images using the Hausdorff distance. IEEE Trans Pattern Anal Mach Intell 15(9):850–863. 10.1109/34.232073

[52] Dennis BR, Tolbert AK (2019) A remarkably narrow RHESSI X-Ray Flare on 2011 September 25. Astrophys J 887(2):131. 10.3847/1538-4357/ab4f81

[53] Massa P, Battaglia AF, Volpara A, Collier H, Hurford GJ, Kuhar M, Perracchione E, Garbarino S, Massone AM, Benvenuto F, Schuller F, Warmuth A, Dickson ECM, Xiao H, Maloney SA, Ryan DF, Piana M, Krucker S (2022) First hard X-ray imaging results by solar orbiter STIX. Sol Phys 297(7):93. 10.1007/s11207-022-02029-x 35891628 10.1007/s11207-022-02029-x PMC9307546

[54] Jules K, Alexander M (2019) basic comparison of python, Julia, Matlab, IDL and java (2019 edition). Technical report, NASA. https://software.nasa.gov/software/GSC-18111-1

[55] Eo T, Shin H, Kim T, Jun Y, Hwang D (2018) Translation of 1D inverse fourier transform of K-space to an image based on deep learning for accelerating magnetic resonance imaging. In: medical image computing and computer assisted intervention - MICCAI 2018, pp. 241–249. Springer, Cham. 10.1007/978-3-030-00928-1_28

[56] Zhu B, Liu JZ, Cauley SF, Rosen BR, Rosen MS (2018) Image reconstruction by domain-transform manifold learning. Nature 555(7697):487–492. 10.1038/nature25988 29565357 10.1038/nature25988

[57] Gossard A, Weiss P (2024) Training adaptive reconstruction networks for blind inverse problems. SIAM J Imag Sci 17(2):1314–1346. 10.1137/23M1545628

[58] Abadi M, Barham P, Chen J, Chen Z, Davis A, Dean J, Devin M, Ghemawat S, Irving G, Isard M, Kudlur M, Levenberg J, Monga R, Moore S, Murray DG, Steiner B, Tucker P, Vasudevan V, Warden P, Wicke M, Yu Y, Zheng X (2016) TensorFlow: A system for large-scale machine learning. In: 12th USENIX Symposium on operating systems design and implementation (OSDI 16), Savannah, GA, USA, pp. 265–283

[59] Virtanen P, Gommers R, Oliphant TE, Haberland M, Reddy T, Cournapeau D, Burovski E, Peterson P, Weckesser W, Bright J, Walt SJ, Brett M, Wilson J, Millman KJ, Mayorov N, Nelson ARJ, Jones E, Kern R, Larson E, Carey CJ, Polat I, Feng Y, Moore EW, VanderPlas J, Laxalde D, Perktold J, Cimrman R, Henriksen I, Quintero EA, Harris CR, Archibald AM, Ribeiro AH, Pedregosa F, Mulbregt P (2020) SciPy 1.0: fundamental algorithms for scientific computing in Python. Nat Methods 17(3):261–272. 10.1038/s41592-019-0686-2 32015543 10.1038/s41592-019-0686-2 PMC7056644

[60] Robitaille TP, Tollerud EJ, Greenfield P, Droettboom M, Bray E, Aldcroft T, Davis M, Ginsburg A, Price-Whelan AM, Kerzendorf WE, Conley A, Crighton N, Barbary K, Muna D, Ferguson H, Grollier F, Parikh MM, Nair PH, Günther HM, Deil C, Woillez J, Conseil S, Kramer R, Turner JEH, Singer L, Fox R, Weaver BA, Zabalza V, Edwards ZI, Bostroem KA, Burke DJ, Casey AR, Crawford SM, Dencheva N, Ely J, Jenness T, Labrie K, Lim PL, Pierfederici F, Pontzen A, Ptak A, Refsdal B, Servillat M, Streicher O (2013) Astropy: a community Python package for astronomy. Astron Astrophys 558:33. 10.1051/0004-6361/201322068

[61] ...Collaboration TA, Price-Whelan AM, Lim PL, Earl N, Starkman N, Bradley L, Shupe DL, Patil AA, Corrales L, Brasseur CE, Nöthe M, Donath A, Tollerud E, Morris BM, Ginsburg A, Vaher E, Weaver BA, Tocknell J, Jamieson W, Kerkwijk MH, Robitaille TP, Merry B, Bachetti M, Günther HM, Aldcroft TL, Alvarado-Montes JA, Archibald AM, Bódi A, Bapat S, Barentsen G, Bazán J, Biswas M, Boquien M, Burke DJ, Cara D, Cara M, Conroy KE, Conseil S, Craig MW, Cross RM, Cruz KL, D’Eugenio F, Dencheva N, Devillepoix HAR, Dietrich JP, Eigenbrot AD, Erben T, Ferreira L, Foreman-Mackey D, Fox R, Freij N, Garg S, Geda R, Glattly L, Gondhalekar Y, Gordon KD, Grant D, Greenfield P, Groener AM, Guest S, Gurovich S, Handberg R, Hart A, Hatfield-Dodds Z, Homeier D, Hosseinzadeh G, Jenness T, Jones CK, Joseph P, Kalmbach JB, Karamehmetoglu E, Kałuszyński M, Kelley MSP, Kern N, Kerzendorf WE, Koch EW, Kulumani S, Lee A, Ly C, Ma Z, MacBride C, Maljaars JM, Muna D, Murphy NA, Norman H, O’Steen R, Oman KA, Pacifici C, Pascual S, Pascual-Granado J, Patil RR, Perren GI, Pickering TE, Rastogi T, Roulston BR, Ryan DF, Rykoff ES, Sabater J, Sakurikar P, Salgado J, Sanghi A, Saunders N, Savchenko V, Schwardt L, Seifert-Eckert M, Shih AY, Jain AS, Shukla G, Sick J, Simpson C, Singanamalla S, Singer LP, Singhal J, Sinha M, Sipőcz BM, Spitler LR, Stansby D, Streicher O, Šumak J, Swinbank JD, Taranu DS, Tewary N, Tremblay GR, Val-Borro M, Van Kooten SJ, Vasović Z, Verma S, Cardoso JVDM, Williams PKG, Wilson TJ, Winkel B, Wood-Vasey WM, Xue R, Yoachim P, Zhang C, Zonca A (2022) The Astropy project: sustaining and growing a community-oriented open-source project and the latest major release (v5.0) of the core package. Astrophys J 935(2):167. 10.3847/1538-4357/ac7c74

[62] Harris CR, Millman KJ, Walt SJ, Gommers R, Virtanen P, Cournapeau D, Wieser E, Taylor J, Berg S, Smith NJ, Kern R, Picus M, Hoyer S, Kerkwijk MH, Brett M, Haldane A, Río JF, Wiebe M, Peterson P, Gérard-Marchant P, Sheppard K, Reddy T, Weckesser W, Abbasi H, Gohlke C, Oliphant TE (2020) Array programming with NumPy. Nature 585(7825):357–362. 10.1038/s41586-020-2649-2 32939066 10.1038/s41586-020-2649-2 PMC7759461

[63] Svd Walt, Schönberger JL, Nunez-Iglesias J, Boulogne F, Warner JD, Yager N, Gouillart E, Yu T (2014) scikit-image: image processing in Python. PeerJ 2:453. 10.7717/peerj.453 10.7717/peerj.453 PMC4081273 25024921

[64] Pedregosa F, Varoquaux G, Gramfort A, Michel V, Thirion B, Grisel O, Blondel M, Prettenhofer P, Weiss R, Dubourg V, Vanderplas J, Passos A, Cournapeau D, Brucher M, Perrot M, Duchesnay E (2011) Scikit-learn: machine learning in python. J Mach Learn Res 12(85):2825–2830

[65] Hunter JD (2007) Matplotlib: a 2D graphics environment. Comput Sci Eng 9(3):90–95. 10.1109/MCSE.2007.55

